# A biosynthetic network for protoberberine production in *Coptis chinensis*

**DOI:** 10.1093/hr/uhad259

**Published:** 2023-12-13

**Authors:** Linrui Wu, Binxin Zhao, Zixin Deng, Bin Wang, Yi Yu

**Affiliations:** Department of Gastroenterology, Zhongnan Hospital of Wuhan University, Hubei Clinical Center and Key Laboratory of Intestinal and Colorectal Disease, School of Pharmaceutical Sciences, Wuhan University, 185 East Lake Road, Wuhan 430071, China; Department of Gastroenterology, Zhongnan Hospital of Wuhan University, Hubei Clinical Center and Key Laboratory of Intestinal and Colorectal Disease, School of Pharmaceutical Sciences, Wuhan University, 185 East Lake Road, Wuhan 430071, China; Department of Gastroenterology, Zhongnan Hospital of Wuhan University, Hubei Clinical Center and Key Laboratory of Intestinal and Colorectal Disease, School of Pharmaceutical Sciences, Wuhan University, 185 East Lake Road, Wuhan 430071, China; State Key Laboratory of Non-Food Biomass and Enzyme Technology, Guangxi Academy of Sciences, Nanning 530007, China; Department of Gastroenterology, Zhongnan Hospital of Wuhan University, Hubei Clinical Center and Key Laboratory of Intestinal and Colorectal Disease, School of Pharmaceutical Sciences, Wuhan University, 185 East Lake Road, Wuhan 430071, China; State Key Laboratory of Non-Food Biomass and Enzyme Technology, Guangxi Academy of Sciences, Nanning 530007, China

## Abstract

Protoberberine alkaloids are a group of tetracyclic isoquinoline compounds known for their well-established antimicrobial and anti-inflammatory properties. The richness and diversity of protoberberine alkaloids accumulated in the *Coptis* genus necessitate a comprehensive examination of the biosynthetic machinery to understand their ecological significance. Here, from *Coptis chinensis* we identified CcCYP719A1, which could install a methylenedioxy bridge on either ring A or ring D of the protoberberine backbone, thus diverging metabolite flux towards the biosynthesis of various protoberberine components. We also obtained CcCYP719A2 and CcCYP719A3, which underwent positive selection after diverging from CcCYP719A1 and maintained specific catalytic activity on ring D. Further, we resolved the biosynthetic pathway of jatrorrhizine by identifying two demethylases, which could also modulate protoberberine composition by removing the C-3 methyl group and methylenedioxy bridge of ring D, allowing demethylated metabolites to be redirected into different routes. Moreover, we characterized 2-*O*-methyltransferase CcOMT1 and flavin-dependent oxidase CcTHBO, respectively responsible for the commonly observed 2-*O*-methylation and aromatic ring-C assembly in protoberberine alkaloids. Overall, this study reveals an interconnected metabolite network from which diverse protoberberine alkaloids originate. It provides valuable insights into the existence of undiscovered protoberberine components, and paves the way for the targeted production of desired protoberberine components for potential therapeutic development.

## Introduction

**Figure 1:**
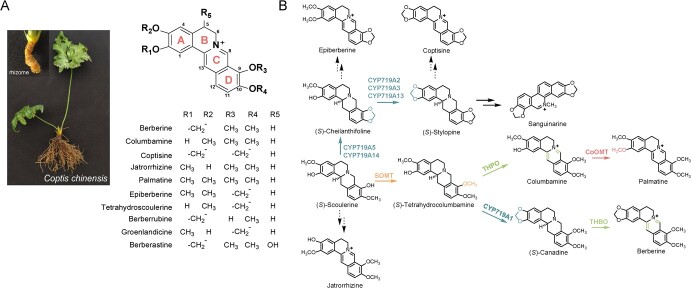
Protoberberine alkaloids produced by *Coptis* genus. **A** Chinese indigenous *Coptis* cultivar *C. chinensis* and representative protoberberine alkaloids derived from Coptidis Rhizoma. **B** Previously elucidated biosynthetic pathway of protoberberine alkaloids. Unverified steps are indicated with dashed arrows.

Throughout history, plants with beneficial bioactivities have been sought after and consumed as medicinal remedies. Ancient pharmacological texts, which documented the usage of herbal medicines, are invaluable resources that have significantly influenced modern pharmacology and the development of novel pharmacophores. A notable example is the discovery of artemisinin, an anti-malaria natural product identified from *Artemisia annua* L., with historical usage dating back over 2000 years for its anti-infective properties, as recorded in the Shennong’s *Classic of Materia Medica* [1, 2]. In addition to *A. annua*, numerous other medicinal herbs with excellent therapeutic properties have been passed down through a thousand years of practice. One distinctive example is Coptidis Rhizoma, generally prepared from the rhizome of *Coptis chinensis* Franch., *C. deltoidea* C. Y. Cheng et Hsiao, or *C. teeta* Wall [3]. Culturally, Coptidis Rhizoma is renowned for its intensely bitter taste and characteristic yellow staining. Pharmacologically, it has been an essential ingredient in many ancient pharmacological recipes for treating inflammation and various gastrointestinal ailments [4]. Modern phytochemical and pharmacological research has revealed that the primary active constituents of Coptidis Rhizoma are a group of benzylisoquinoline alkaloids (BIAs) featuring the 5,6-dihydrodibenzo[a,g]quinolizinium system, collectively known as protoberberine alkaloids (Fig. 1A) [5, 6]. Although protoberberine alkaloids are broadly found in various plant families, such as Ranunculaceae, Papaveraceae, Berberidaceae, Menispermaceae, and Rutaceae, species of the *Coptis* genus appear to be outstanding producers of protoberberine alkaloids in terms of quantity and diversity [4, 7]. Over 20 protoberberine components have been found in *Coptis* species, the total content ranging from 5 to 10% [8, 9]. Yet our knowledge about the physiological role of protoberberine alkaloids in the survival and fitness of *Coptis* species remains limited. The ecological significance of the lineage-specific increase in the abundance and diversity of protoberberine is also unknown. Addressing these questions would require a comprehensive understanding of protoberberine alkaloid biosynthesis.

**Figure 2:**
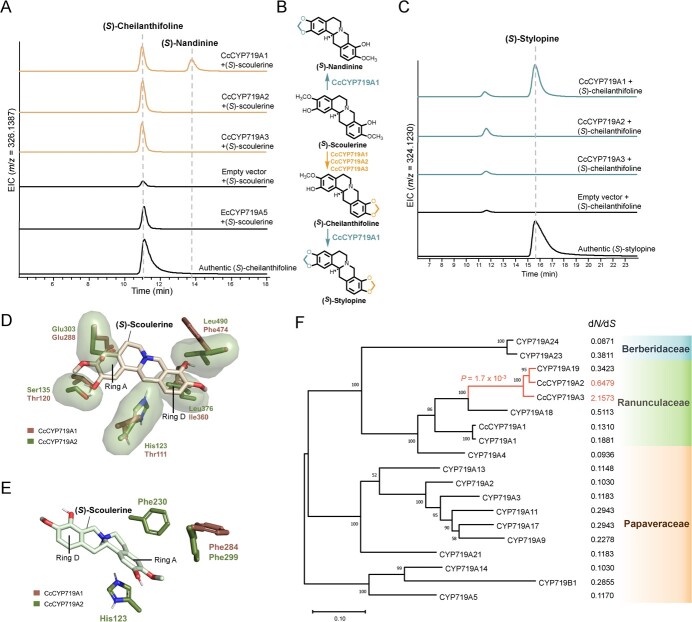
Functional characterization of CYP719 enzymes for coptisine and epiberberine biosynthesis. **A** CYP719 enzyme assays using (*S*)-scoulerine as substrate. Extracted ion chromatograms corresponding to the theoretical *m*/*z* value of (*S*)-cheilanthifoline and (*S*)-nandinine. EcCYP719A5 was used as the positive control. **B** Transformation of (*S*)-scoulerine towards (*S*)-nandinine and (*S*)-stylopine, respectively, catalyzed by CYP719 enzymes characterized from *C. chinensis*. **C** CYP719 enzyme assays using (*S*)-cheilanthifoline as the substrate. Extracted ion chromatograms corresponding to the theoretical *m*/*z* value of (*S*)-stylopine. **D** Structural modeling of CcCYP719A1 (brown) and CcCYP719A2 (green). (*S*)-Scoulerine is shown in the conformation of binding in CcCYP719A1. The active site of CcCYP719A1 is more spacious than that of CcCYP719A2. **E** His123, Phe230, and Phe299 in CcCYP719A2 might contribute to substrate positioning through π–π stacking interactions. (*S*)-Scoulerine is shown in the conformation of binding in CcCYP719A2. **F** Maximum likelihood phylogenetic tree of sequences of CYP719A family, labeled with associated d*N*/d*S* values and phylogenetic origin. The scale bar indicates substitutions per site. Bootstrap values from 1000 replicates are indicated at each tree node. Foreground lineage used in the test of positive selection is marked in red with the *P* value.

Six major protoberberine alkaloids have been identified in *C. chinensis*, namely berberine, columbamine, palmatine, epiberberine, coptisine, and jatrorrhizine [5]. Among them, the biosynthetic pathway of berberine has been elucidated (Fig. 1B). (*S*)-Scoulerine, the common precursor of protoberberine alkaloids, first undergoes 9-*O*-methylation by (*S*)-scoulerine 9-*O*-methyltransferase (SOMT) to generate (*S*)-tetrahydrocolumbamine [10], which is then subjected to methylenedioxy bridge installation by CYP719A1 (canadine synthase) [11]. Finally, berberine is produced from (*S*)-canadine through oxidation catalyzed by tetrahydroberberine oxidase (THBO) [12, 13]. On this basis, the biosynthetic pathway of columbamine was determined by identifying the oxidation of (*S*)-tetrahydrocolumbamine to generate columbamine by (*S*)-tetrahydroprotoberberine oxidase (THPO) [14]. Further, the biosynthetic pathway for palmatine was determined by characterizing columbamine *O*-methyltransferase, which catalyzes the conversion of columbamine to palmatine (Fig. 1B) [15]. In contrast, both coptisine and epiberberine contain a methylenedioxy bridge on ring D of the protoberberine backbone, distinct from the methylenedioxy bridge of berberine on ring A, suggesting another CYP719 enzyme is responsible for their biosynthesis. The elucidation of the sanguinarine biosynthetic pathway showed that two CYP719 enzymes, cheilanthifoline synthase (CYP719A5) and stylopine synthase (CYP719A2), identified from *Eschscholzia californica*, convert (*S*)-scoulerine into (*S*)-stylopine [16, 17]. It is likely that epiberberine and coptisine are respectively derived from (*S*)-cheilanthifoline and (*S*)-stylopine, and their complete biosynthetic pathways are yet to be established (Fig. 1B, dashed arrows). An exhaustive search for the potential methylenedioxy bridge-installing enzyme from the *C. chinensis* genome resulted in the identification of six candidates [18, 19]. Subsequent biochemical assays only determined one candidate (Cch00017825) that can install a methylenedioxy bridge on ring A, leaving the putative CYP719 enzyme for ring D unaccounted for [19]. Compared with the above-mentioned protoberberine alkaloids, jatrorrhizine lacks an *O*-methylation modification on the hydroxyl group of C-3, which is already installed on (*S*)-scoulerine. This discrepancy has raised the question of whether (*S*)-scoulerine is a biosynthetic precursor for jatrorrhizine. Surrounding this, various hypotheses have been made with regard to the branching point from which the biosynthesis of jatrorrhizine diverges [20, 21]. However, to date, none of these hypotheses has been validated through the identification of corresponding biosynthetic enzymes or intermediates from jatrorrhizine-producing plants.

Here we unveil a biosynthetic network for protoberberine alkaloids in *C. chinensis*. We demonstrate that the synthetic capacity of this network surpasses the currently established phytochemical profile of *C. chinensis*, suggesting the potential application of the characterized enzymes in alternative methods to access valuable protoberberine components.

## Results

### Versatile CcCYP719A1 is primarily responsible for protoberberine biosynthesis

We first obtained the transcriptome of *C. chinensis* by subjecting freshly sampled leaf, root, and rhizome to RNA sequencing. This generated 820.2 million clean reads, from which we assembled a transcriptome containing 94 916 unique transcripts. The expression level of the transcripts was quantified and normalized across the samples. Both coptisine and epiberberine contain a methylenedioxy bridge structure, a modification generally installed by enzymes of the CYP719 clan in the plant’s specialized metabolism [11]. Therefore, we examined the expression level of the six candidate CYP719 transcripts (referred to as *CcCYP719A1* to *CcCYP719A6*; Supplementary Data Table S1). Among them, *CcCYP719A1* showed the highest expression level, while the remaining five transcripts were barely expressed in all sequenced samples (Supplementary Data Fig. S1). To confirm the activity of the CYP719 candidates, all six transcripts were cloned onto the pESC-HIS vector and expressed individually in *Saccharomyces cerevisiae* strain WAT11, which incorporates the *Arabidopsis thaliana* origin cytochrome P450 reductase *ATR1* [22]. Microsomes were prepared from the recombinant WAT11 strain, and subjected to enzyme assay using (*S*)-scoulerine as substrate. The cheilanthifoline synthase CYP719A5, identified from *E. californica*, was used as control. Among the candidates, CcCYP719A1 (Cch00017825) transformed the substrate into (*S*)-nandinine as previously reported [19]. The MS^2^ spectrum of the hypothesized (*S*)-nandinine showed peaks at 176.0706 and 151.0754, representing free hydroxyl and methoxy groups on ring D and the methylenedioxy bridge on ring A, indicating it is (*S*)-nandinine (Supplementary Data Fig. S2). Surprisingly, we found that CcCYP719A1, CcCYP719A2, and CcCYP719A3 could also convert (*S*)-scoulerine into (*S*)-cheilanthifoline, compared with the retention time of (*S*)-cheilanthifoline standard (Fig. 2A). The MS^2^ spectrum of both the (*S*)-cheilanthifoline product and the standard compound exhibited distinctive peaks at 178.0863 and 149.0597, indicating a free hydroxyl and methoxy group on ring A and a methoxy group on ring D (Supplementary Data Fig. S2). Furthermore, CcCYP719A1 exhibited activity towards (*S*)-cheilanthifoline and (*S*)-tetrahydrocolumbamine, producing (*S*)-stylopine and (*S*)-canadine, respectively (Fig. 2B and C, Supplementary Data Fig. S3). However, CcCYP719A2 and CcCYP719A3 showed no such activity. These findings demonstrate that CcCYP719A1 possesses cheilanthifoline synthase, stylopine synthase, and canadine synthase activities, indicating its ability to catalyze methylenedioxy bridge formation on both ring A and ring D of the protoberberine backbone.

CcCYP719A1 exhibits a high sequence identity of ~70% with CcCYP719A2/CcCYP719A3, but it demonstrated greater substrate promiscuity compared with its two paralogs. To investigate the structural basis for their different substrate selectivity, we employed AlphaFold to predict the 3D structures of both CcCYP719A1 and CcCYP719A2. Employing molecular docking analysis, we discovered that (*S*)-scoulerine could bind in two distinct orientations in the catalytic pocket of CcCYP719A1 with similar binding energy (Supplementary Data Fig. S4). On the other hand, (*S*)-scoulerine adopts a slanted position in CcCYP719A2, with its hydroxyl group and methoxy group of ring D directed towards the catalytic heme (Supplementary Data Fig. S4). Notably, the active site of CcCYP719A1 contains residues such as Thr111, Thr120, Glu288, Ile360, and Phe474, which, when compared with their counterparts in CcCYP719A2 (His123, Ser135, Glu303, Leu376, and Leu490), offer less steric hindrance to the protoberberine scaffold, resulting in greater substrate binding flexibility (Fig. 2D). This flexibility allows the substrate to orient either ring A or ring D towards the catalytic heme, accounting for the observed substrate promiscuity of CcCYP719A1. Moreover, His123, Phe230, and Phe299 in CcCYP719A2 appear to play a crucial role in positioning and stabilizing the substrate through π–π stacking interactions with the tetracyclic ring. However, the corresponding residues in CcCYP719A1 (Ile215, Phe284, and Thr111) are unable to achieve the same effect (Fig. 2E). Additionally, the modeling of (*S*)-cheilanthifoline and (*S*)-tetrahydrocolumbamine in CcCYP719A2 displayed a similar slanted conformation, with their respective ring D positioned towards the catalytic site. Taken together, these factors exclude the potential activity of CcCYP719A2 on ring A of protoberberine alkaloid, resulting in its specific cheilanthifoline synthase activity (Supplementary Data Fig. S4).

Genomic information reported earlier demonstrated that *CcCYP719A4*–*CcCYP719A6* are clustered with *CcCYP719A1* on chromosome 3, suggesting that they might have originated from a tandem duplication event. In contrast, *CcCYP719A2* and *CcCYP719A3* are located on chromosomes 4 and 9, respectively [19]. This arrangement implies that *CcCYP719A2* and *CcCYP719A3* may have originated from a whole-genome duplication (WGD) event. To further explore the evolutionary relationships of the characterized CYP719 enzymes in this study, a maximum likelihood tree was constructed using multiple sequence alignments of CYP719 genes identified from various plant families (Fig. 2F). *CcCYP719A1* shares close phylogeny with its ortholog *CYP719A1* from *Coptis japonica*. However, a previous study as well as our own enzyme assays have indicated that CYP719A1 only exhibits canadine synthase activity (Supplementary Data Fig. S5) [11]. The different substrate scopes exhibited by CcCYP719A1 and CYP719A1 suggests that slight variations in the primary sequence of CYP719 enzymes can shift the substrate recognition pattern. Interestingly, *CYP719A18* and *CYP719A19* from *C. japonica*, which belong to a distinct clade in the phylogenetic tree, showed close phylogeny with *CcCYP719A2* and *CcCYP719A3*. Consistent with this close phylogeny, biochemical characterization of CYP719A18 and CYP719A19 revealed that they also possess cheilanthifoline synthase activities, but are not active towards (*S*)-tetrahydrocolumbamine or (*S*)-cheilanthifoline (Supplementary Data Fig. S6). The close phylogeny of *CcCYP719A2* and *CcCYP719A3* with *CYP719A18* and *CYP719A19*, and their identical activity, indicate that the two pairs of orthologs diverged and subfunctionalized from their respective paralogs *CcCYP719A1* and *CYP719A1* before the divergence of *C. japonica* and *C. chinensis*. It has been reported that *C. chinensis* and *A. coerulea* share a WGD event, which played an important role in the diversification of CYP genes [18, 19]. *CcCYP719A2* and *CcCYP719A3* may have also originated from this WGD event, and further evolutionary analysis is needed to determine the evolutionary trajectory of *CcCYP719A2* and *CcCYP719A3.* As for *C. japonica*, since all three characterized CYP719 enzymes showed no stylopine synthase activity (Supplementary Data Figs S5 and S6) [11], there should exist unidentified enzymes responsible for coptisine biosynthesis.

As shown, both *CcCYP719A2* and *CcCYP719A3* are expressed at minimal level in all sequenced samples, and their biochemical function could be fully covered by CcCYP719A1 (Fig. 2, Supplementary Data Fig. S1). This led us to hypothesize that in *C. chinensis* only CcCYP719A1 is actively involved in protoberberine biosynthesis. To estimate the selection pressure imposed on the CYP719-encoding genes that might reflect their physiological function, non-synonymous to synonymous substitution rates (ω = *d*_N_*/d*_S_) were calculated. The lineage-specific *d*_N_*/d*_S_ ratios (ω values) suggest that, among all other CYP719-encoding genes, *CcCYP719A2* and *CcCYP719A3* are under positive selection, particularly with the ω value of CcCYP719A3 being >1. In contrast, the low ω values for both CcCYP719A1 and CYP719A1 indicate that they are under more stringent constraints than their paralogs, likely due to their roles in berberine biosynthesis [23]. To further investigate selection pressures, a branch-site analysis was conducted on the branch containing *CcCYP719A2*, *CcCYP719A3*, and *CYP719A19*, with the remaining phylogenetic tree treated as the background lineage [24]. The analysis revealed significant positive selection on this branch (Fig. 2E, Supplementary Data Table S2). Considering that CcCYP719A2 and CcCYP719A3 can be functionally covered by CcCYP719A1 and they are minimally expressed in all sequenced samples, it is plausible that these genes have experienced a relaxation of evolutionary constraints since diverging from CcCYP719A1. Mutations accumulated since the divergence of *CcCYP719A1* and *CcCYP719A2*/CcCYP719A3 could have resulted in the subfunctionalization of *CcCYP719A2* and *CcCYP719A3*. Overall, these findings further imply the indispensable role of CcCYP719A1 in protoberberine biosynthesis.

### Biosynthetic pathways of epiberberine and coptisine

**Figure 3:**
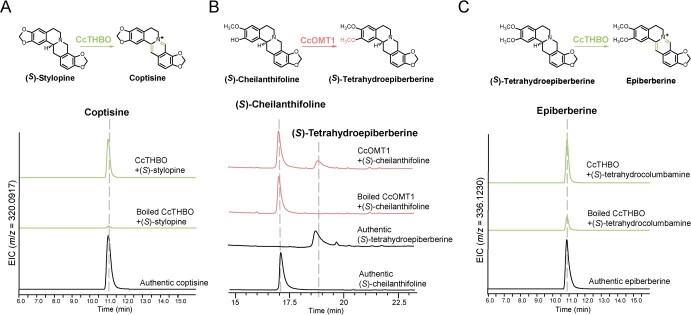
Characterization of epiberberine and coptisine biosynthetic pathways. **A** Enzyme assay of CcTHBO using (*S*)-stylopine as substrate to produce coptisine. Extracted ion chromatograms correspond to the theoretical *m*/*z* value of coptisine. **B** HPLC analysis of the *in vitro* assay of CcOMT1. Incubation of CcOMT1 with (*S*)-cheilanthifoline generated (*S*)-tetrahydroepiberberine. Boiled protein was used as the negative control. **C** Enzyme assay of CcTHBO using (*S*)-tetrahydroepiberberine as substrate to produce epiberberine. Extracted ion chromatograms correspond to the theoretical *m*/*z* value of epiberberine.

To complete the biosynthesis of coptisine from (*S*)-stylopine, ring C of the protoberberine backbone needs to be oxidized to form protoberberinium salt. This reaction has been previously reported to be catalyzed by the flavin-dependent THBO [12, 25]. To identify the corresponding oxidase involved in coptisine biosynthesis, we conducted a local BLASTP search using THBO-encoding genes characterized from *C. japonica* (CjTHBO), *Berberis wilsoniae* (BwTHBO), and *Argemone mexicana* (AmTHBO) as query sequences against the *C. chinensis* transcriptome. Coupled with a Pfam domain search for the FAD binding domain (PF01565) and berberine domain (PF08031), 13 candidate transcripts were identified, which were subjected to phylogenetic analysis with THBOs, berberine bridge enzymes (BBEs), tetrahydrocannabinolic acid synthase (THCA) and *Cannabis sativa*-derived cannabidiolic acid synthase (CBDA). Among the candidates, only one transcript (*CcTHBO*) clustered with other characterized THBOs and also exhibited a high expression level in root and rhizome samples (Supplementary Data Fig. S7). As the overexpression and purification of CcTHBO failed in both *E. coli* and *S. cerevisiae* systems, we employed *Pichia pastoris* strain GS115 (Invitrogen) as the expression host. The recombinant protein obtained was incubated with (*S*)-stylopine, resulting in the production of coptisine, which was confirmed by LC–MS analysis (Fig. 3A, Supplementary Data Fig. S8). Additionally, we tested the activity of CcTHBO against (*S*)-canadine and (*S*)-tetrahydrocolumbamine, resulting in the production of berberine and columbamine, respectively (Supplementary Data Fig. S9).

For epiberberine biosynthesis, a methyltransferase edna is required to install a methyl group onto the hydroxyl group at C-2. To identify the relevant methyltransferase involved in this process, we conducted a BLASTP search using 15 characterized methyltransferases responsible for BIA biosynthesis as query sequences against the transcriptome of *C. chinensis*. This search yielded 80 candidate transcripts. Further, we excluded transcripts with coding sequences shorter than 600 bp, resulting in 38 remaining candidates. These candidates were then subjected to phylogenetic analysis using representative sequences from three methyltransferase families (Supplementary Data Fig. S10) [26]. With the 14 candidates that clustered with known BIA biosynthetic genes, we performed Pfam domain analysis and multiple sequence alignment. Transcripts whose encoded proteins lack a conserved histidine residue essential for forming a hydrogen bond with the target hydroxyl group were excluded (Supplementary Data Fig. S11) [27]. The remaining 10 candidates were manually curated to exclude transcripts with >90% identity to characterized methyltransferases, which are responsible for other methylation steps in BIA biosynthesis. Finally, three transcripts were selected for further characterization based on their overall expression levels, designated as *CcOM1* to *CcOMT3* (Supplementary Data Fig. S12). The selected candidates were cloned and overexpressed in *E. coli* for enzymatic assay. Among them, CcOMT1 exhibited activity towards (*S*)-cheilanthifoline, resulting in the production of (*S*)-tetrahydroepiberberine (Fig. 3B). (*S*)-Tetrahydroepiberberine can also be transformed by CcTHBO into epiberberine, thereby establishing its biosynthetic pathway (Fig. 3C). Alternatively, we tested CcOMT1 on (*S*)-scoulerine, and found that it produced a methylation product with different retention time from (*S*)-tetrahydrocolumbamine, suggesting that the methylation occurs on the hydroxyl group of C-2 instead of C-9. Further, we prepared methylated scoulerine and used it as a substrate in the enzyme assay of CcCYP719A1, which resulted in the production of (*S*)-tetrahydroepiberberine (Supplementary Data Fig. S13). This confirms that, for epiberberine biosynthesis, enzyme promiscuity allows the reaction sequences of methylation and methylenedioxy bridge formation to be interchangeable. Overall, the biosynthetic pathways of coptisine and epiberberine are established by identifying the versatile CcCYP719A1, which directs the branching from (*S*)-scoulerine to generate products with methylenedioxy bridge modification on ring D.

### Biosynthetic pathway of jatrorrhizine

(*S*)-Scoulerine, constructed by the berberine bridge enzyme from (*S*)-reticuline, is the common precursor of protoberberine alkaloids. (*S*)-Scoulerine is methylated on the C-3 hydroxyl group, and this modification is present in all major protoberberine alkaloids produced in *C. chinensis* except for jatrorrhizine. This discrepancy therefore becomes the most critical issue in elucidating jatrorrhizine biosynthesis, regarding whether (*S*)-scoulerine is a precursor of jatrorrhizine. If (*S*)-scoulerine is not a precursor of jatrorrhizine, the biosynthesis of jatrorrhizine must have diverged from the isoquinoline common pathway as early as norcoclaurine, which is the condensation product of dopamine and 4-hydroxyphenylacetaldehyde (Supplementary Data Fig. S14). This indicates that downstream tailoring enzymes should accept the unmethylated substrates in parallel to serve for jatrorrhizine biosynthesis. However, no unmethylated intermediate derived from (*S*)-norcoclaurine has been reported, leaving this assumption weakly supported. Alternatively, if (*S*)-scoulerine is indeed the precursor of jatrorrhizine, a demethylase is required to remove the *O*-methylation on C-3 at some point along the pathway. In fact, enzymes from several families have been reported to catalyze demethylation reactions [28–31]. Notably, the biosynthesis of morphine, another type of BIA, also involves demethylation steps. Its biosynthetic intermediates thebaine and codeine can be transformed by codeine *O*-demethylase (CODM), a 2-oxoglutarate/Fe(II)-dependent dioxygenase, to produce oripavine and morphine, respectively [32]. Based on this, we conducted a BLAST search against the *C. chinensis* transcriptome using CODM from *Papaver somniferum* as query sequence. Three candidates were identified with moderate amino acid sequence identity to CODM (~40–50%). All demethylase candidates, as well as CODM from *P. somniferum* as control, were purified as recombinant protein from an *E. coli* expression system for characterization.

**Figure 4:**
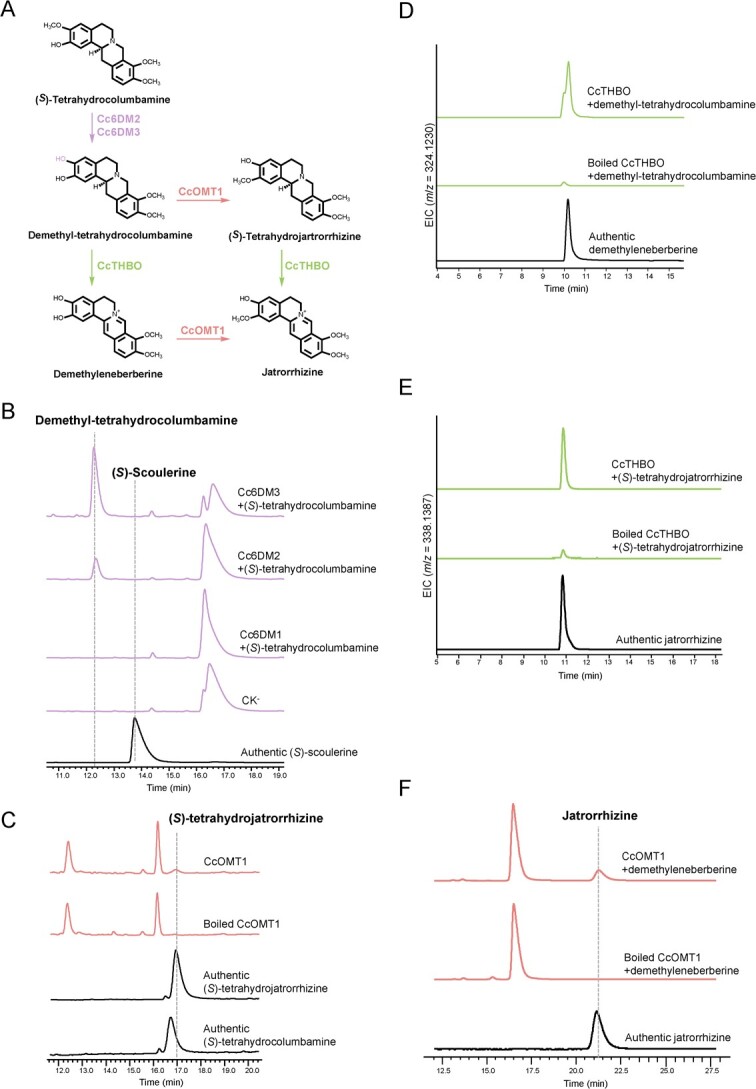
Characterizing the biosynthetic pathway for jatrorrhizine. **A** Characterized biosynthetic pathway for jatrorrhizine derived from the demethylation of (*S*)-tetrahydrocolumbamine. **B** HPLC analysis showing the demethylation of (*S*)-tetrahydrocolumbamine in the enzyme assays of candidate demethylases. **C** HPLC analysis of CcOMT1 assay using demethyl-tetrahydrocolumbamine as substrate, showing the production of (*S*)-tetrahydrojatrorrhizine. **D** Enzyme assay of CcTHBO using demethyl-tetrahydrocolumbamine to produce demethyleneberberine. Extracted ion chromatograms corresponding to the theoretical *m*/*z* value of demethyleneberberine. **E** Enzyme assay of CcTHBO using (*S*)-tetrahydrocolumbamine as substrate to produce jatrorrhizine. Extracted ion chromatograms corresponding to the theoretical *m*/*z* value of jatrorrhizine. **F** HPLC analysis of the transformation from demethyleneberberine to jatrorrhizine under catalysis by CcOMT1.

To determine the substrate of the candidates Cc6DM1–Cc6DM3, we conducted assays using protoberberine components lacking the methylenedioxy bridge modification: (*S*)-scoulerine, (*S*)-tetrahydrocolumbamine, columbamine, and palmatine. When (*S*)-scoulerine was used as the substrate, CODM successfully produced the expected demethylated scoulerine, confirming the reliability of our bioassay system (Supplementary Data Fig. S15). Interestingly, another demethylation product was observed in the reaction mixture of Cc6DM2 and Cc6DM3. This product shares the same molecular mass as the demethylated scoulerine generated by CODM, but showed a slightly earlier elution time in the HPLC analysis, indicating it was likely the C-10 demethylated product of (*S*)-scoulerine (Supplementary Data Fig. S15). When (*S*)-tetrahydrocolumbamine was incubated with Cc6DM2 or Cc6DM3, a demethylation product different from (*S*)-scoulerine was produced; the MS^2^ spectrum of the demethylation product indicates that the demethylation occurs on C-3 (Fig. 4A and B, Supplementary Data Fig. S16). However, none of the demethylase candidates showed activity towards columbamine (Supplementary Data Fig. S17). In the case of palmatine, only a trace amount of transformation was observed in the overnight reaction mixture of Cc6DM2 or Cc6DM3. The retention time and MS^2^ spectrum of the product matched that of authentic jatrorrhizine (Supplementary Data Fig. S18). Since only a trace amount of palmatine was converted into jatrorrhizine, it is likely that palmatine is not the natural substrate for the demethylases. Overall, these results strongly suggest that jatrorrhizine biosynthesis branches from the demethylation of (*S*)-tetrahydrocolumbamine. To be converted to jatrorrhizine, demethyl-tetrahydrocolumbamine needs to be methylated on C-2 and oxidized on ring C. Based on the assembly logic of epiberberine, we propose that CcOMT1 and CcTHBO are responsible for the transformation from demethyl-tetrahydrocolumbamine to jatrorrhizine. Subsequently, we purified demethyl-tetrahydrocolumbamine from the Cc6DM3 assay and tested it with both CcTHBO and CcOMT1. The results showed that both enzymes could utilize demethyl-tetrahydrocolumbamine as substrate, producing (*S*)-tetrahydrojatrorrhizine (also known as corypalmine) and demethyleneberberine, respectively (Fig. 4C and D). Further oxidation of (*S*)-tetrahydrojatrorrhizine by CcTHBO and methylation of demethyleneberberine by CcOMT1 both led to the generation of jatrorrhizine, as confirmed by comparison with an authentic jatrorrhizine (Fig. 4E and F). This demonstrates that the reaction sequence of ring-C oxidation and C-3 methylation can be interchangeable for jatrorrhizine biosynthesis, similar to the biosynthetic routes to (*S*)-tetrahydroepiberberine (Fig. 4A).

It is intriguing why berberine is much more abundant than palmatine and jatrorrhizine in the rhizome of *Coptis* species, as their biosyntheses share the common precursor (*S*)-tetrahydrocolumbamine. We initially hypothesized that columbamine and jatrorrhizine could be transformed into berberine, thus converging the metabolite flux. However, when we tested columbamine and jatrorrhizine with CcCYP719A1, no transformation activity was observed. Testing (*S*)-tetrahydrojatrorrhizine with CcCYP719A1, on the other hand, did produced (*S*)-canadine (Supplementary Data Fig. S19). This finding suggests that a portion of the metabolite flux towards jatrorrhizine could be hijacked by CcCYP719A1 at the stage of (*S*)-tetrahydrojatrorrhizine towards berberine biosynthesis.

### Protoberberine biosynthetic network in *C. chinensis*

**Figure 5:**
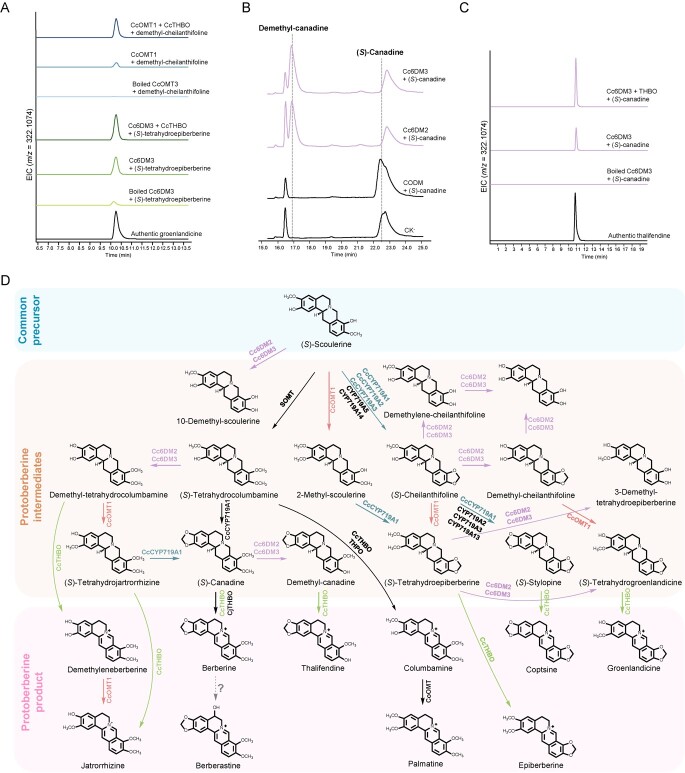
Exploring the synthetic potential of the protoberberine metabolite network. **A** Transformation from demethyl-cheilanthifoline and (*S*)-tetrahydroepiberberine to groenlandicine under catalysis by CcOMT1/CcTHBO, and Cc6DM3/CcTHBO, respectively. Extracted ion chromatograms corresponding to the theoretical *m*/*z* value of groenlandicine. **B** HPLC analysis of the demethylation of (*S*)-canadine, catalyzed by Cc6DM2 and Cc6DM3. **C** Transformation from (*S*)-canadine to thalifendine catalyzed by Cc6DM3 and CcTHBO. Extracted ion chromatograms corresponding to the theoretical *m*/*z* value of thalifendine. **D** Schematic representation of the protoberberine metabolite network characterized in *C. chinensis*. Black arrows indicate reactions characterized in previous studies and colored arrows represent reactions characterized in this study. The dotted arrow indicates proposed 5-hydroxylation to produce berberastine. The blue panel indicates the common precursor (*S*)-scoulerine, the orange panel indicates the protoberberine intermediates characterized from the elucidation of protoberberine biosynthetic pathways, and the pink panel indicates the biosynthetic protoberberinium salt products.

The substrate promiscuity shown by the enzymes characterized in this study implies that *C. chinensis* uses this limited number of enzymes to synthesize a protoberberine repository with great diversity, constituting an interconnected metabolite network. While investigating the phytochemical profile of *C. chinensis*, we noticed that various minor protoberberine alkaloids are produced with similar modifications. We wonder if the assembly logic of protoberberine alkaloids that has been established thus far could be used to further infer the biosynthetic pathway of these minor products. As an example, we proposed the biosynthetic pathway for groenlandicine, a minor protoberberine alkaloid with the same modifications on ring A as jatrorrhizine and the methylenedioxy bridge modification on ring D [4]. To verify its biosynthetic route, we first tested the feasibility of demethylating (*S*)-cheilanthifoline. As a result, three *O*-demethylation and *O*,*O*-demethylenation products were generated from the assays using Cc6DM2 or Cc6DM3 (Supplementary Data Fig. S20). The demethylation and demethylenation activity shown by Cc6DM2 and Cc6DM3 suggests that they could remove modifications on many metabolites from the protoberberine metabolite network, allowing them to be redirected into other biosynthetic routes. Next, the demethylation product of (*S*)-cheilanthifoline was prepared and tested with CcOMT1, which produced a C-2 methylated product (Supplementary Data Fig. S21). This methylation product can then undergo oxidation by CcTHBO, ultimately leading to the formation of groenlandicine (Fig. 5A). Alternatively, (*S*)-tetrahydroepiberberine was also observed to be a viable substrate for Cc6DM2 and Cc6DM3 (Supplementary Data Fig. S22). Incubating (*S*)-tetrahydroepiberberine with both Cc6DM3 and CcTHBO finally generated groenlandicine as expected (Fig. 5A).

**Figure 6:**
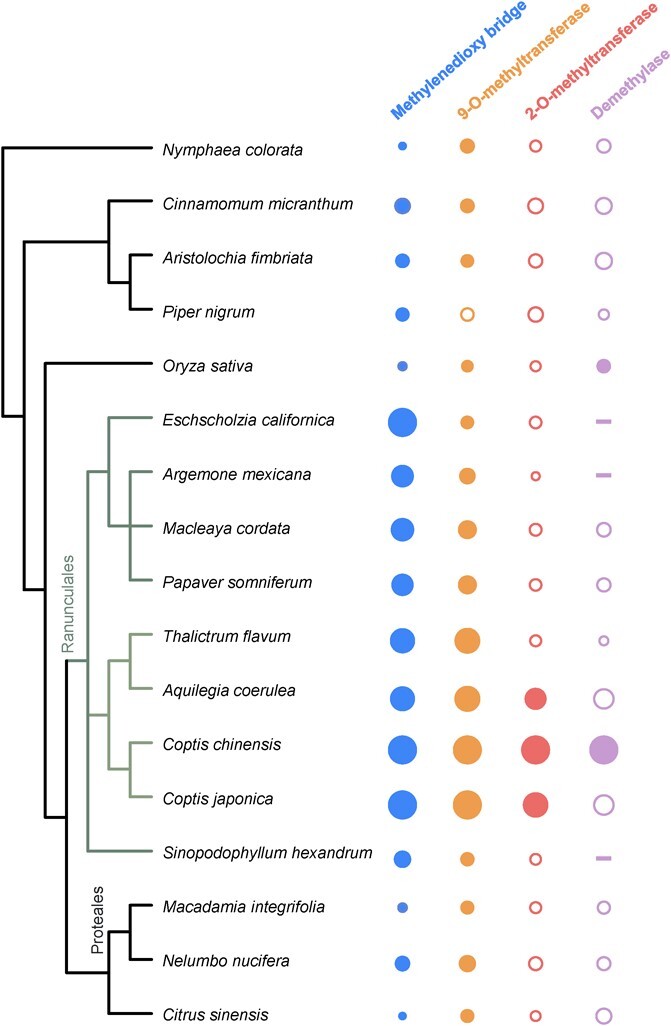
Phylogenetic distribution of the four types of protoberberine modifying enzymes. The phylogenetic tree was created using PhyloT (https://phylot.biobyte.de). The circles are scaled by the amino acid sequence identity of best hit from BLASTP search using CcCYP719A1, CjSOMT, CcOMT1, and Cc6DM3 with the respective plant proteome. Filled circles denote that the homolog returned the respective *C. chinensis* protein in reciprocal BLAST (orthologs identified). Dashes signify that no significant hit was returned by the BLAST search.

It is likely that the protoberberine metabolite network we identified in this study possesses synthetic capabilities that extend beyond the limited range of protoberberine known thus far. This leads us to hypothesize that a broader array of diverse protoberberine alkaloids could be produced by *C. chinensis*. At this point, we noticed that the biosynthesis of thalifendine, a protoberberine alkaloid with a methylenedioxy bridge formed on ring A and methylation on C-9, has not been confirmed in *C. chinensis* [8, 33]. We propose that its synthetic route could be constructed *in vitro* using the characterized biosynthetic enzymes. To investigate this, we initially tested Cc6DM2 and Cc6DM3 on berberine. HPLC analysis revealed that Cc6DM2 catalyzed the production of only trace amounts of thalifendine, as confirmed by comparison with an authentic standard (Supplementary Data Fig. S23). On the other hand, under catalysis by Cc6DM2 and Cc6DM3, (*S*)-canadine could undergo demethylation, and the resulting demethylation product could be further oxidized to yield thalifendine when the reaction system was supplemented with CcTHBO (Fig. 5B and C, Supplementary Data Fig. S24). These findings provide further evidence for the ability of the characterized enzymes to generate a diverse range of protoberberine alkaloids, suggesting the existence of more undiscovered protoberberine alkaloids produced by *C. chinensis* (Fig. 5D).

### Protoberberine biosynthetic genes among benzylisoquinoline alkaloid-producing lineages

The established molecular mechanism from which the chemical diversity of protoberberine alkaloids emerges prompted us to determine whether the identified genes are conserved in protoberberine-producing plants. To analyze the phylogenetic distribution of four types of protoberberine modification enzymes, we conducted a BLAST search to compare the amino acid percentage identity between these enzymes and their closest homologs in 16 different species. Next, we performed reverse BLAST of the identified homologs back to the proteome of *C. chinensis* to identify ortholog protein pairs, which derived from a common progenitor enzyme after the speciation event [34, 35]. As shown in Fig. 6, orthologs of CcCYP719A1 and SOMT are commonly found in Ranunculales species, aligning with the prevalence of berberine production within this order. CYP719 enzymes can also be found in Proteales species *Nelumbo nucifera* and Piperales species *Aristolochia fimbriata*, both of which produce aporphine alkaloids with a methylenedioxy bridge structure [36–39]. It is highly likely that the respective CYP719 enzymes are involved in their biosynthesis, suggesting that the emergence of the CYP719 clan predates the divergence of Ranunculales. *Nelumbo nucifera* and *A. fimbriata* may also produce protoberberine alkaloids that are yet to be identified. The orthologs of SOMT can be found in basal lineages such as Nymphaeales, where BIA biosynthesis has not been established. This indicates that the progenitor of SOMT may have first played a role in BIA biosynthesis as norcoclaurine-6-*O*-methyltransferase (Supplementary Data Fig. S14). In contrast, 2-*O*-methyltransferase (CcOMT1) and demethylases (Cc6DM2/Cc6DM3) appear to have been newly recruited, parallel to the increase in both the quantity and diversity of protoberberine alkaloids in *Coptis* genus. The prosperity of protoberberine alkaloids may have served as an adaptive strategy to enhance the fitness of the *Coptis* genus in cold and moist environments.

## Discussion

*Coptis* species are naturally distributed in diverse environments ranging from warm temperate to cold coniferous forests [40, 41]. In these mild and humid habitats, favorable conditions promote the growth of diverse microorganisms that actively interact with protoberberine alkaloids released by *Coptis* plants through root exudates [42, 43]. The enrichment of protoberberine alkaloids in underground organisms might also play a crucial role in shaping soil composition and the surrounding plant community, facilitating the adaptation of *Coptis* species to their ecological niches [44, 45]. Understanding the ecological significance of producing such a complex array of protoberberine alkaloids requires a detailed investigation of the molecular mechanisms of their biosynthesis. In this study, we characterized the biosynthetic pathway of key protoberberine components in *C. chinensis*, revealing a complex and interconnected metabolite network (Fig. 5D). Our findings demonstrate that the diversity of protoberberine alkaloids does not arise from the actions of specific enzymes dedicated to distinct routes, but is achieved through the collaboration of versatile modifying enzymes. These enzymes catalyze branching reactions from various biosynthetic intermediates, ultimately giving rise to different protoberberine components. This protoberberine metabolite network holds significant promise for the targeted biosynthesis of protoberberine components and their derivatives in selected chassis cells. By manipulating the expression of the corresponding biosynthetic enzymes, we could potentially engineer the production of specific protoberberine alkaloids with tailored properties. Furthermore, the protoberberine metabolite network not only delineates the biosynthetic routes of major protoberberine components but also establishes the underlying assembly logic for the entire group of protoberberine alkaloids. This valuable insight allows us to predict the biosynthetic routes of uncharacterized protoberberine components by tracing relevant intermediates through the metabolite network. For example, C-5 hydroxylated berberine (berberastine), which is also produced by *C. chinensis*, has not been biosynthetically characterized thus far [4, 46]. However, its biosynthetic pathway could be readily inferred from the network, which may branch from the berberine biosynthetic route (Fig. 5D).

In exploring plant specialized metabolism, poorly accumulated metabolites might elude detection due to limitations in analytical methodologies. This raises the intriguing question of whether there are hidden protoberberine components beyond the currently established phytochemical profile of *C. chinensis*. The metabolite network has shed light on this hidden protoberberine repository, as demonstrated by the *in vitro* reconstitution of thalifendine biosynthesis (Fig. 5B and C). This implies that the biosynthetic route of some unidentified protoberberine components, represented by thalifendine, is diminished under physiological conditions by unknown regulatory mechanisms, hindering their characterization. Consequently, it is likely that the actual biosynthetic capacity of *C. chinensis* has been underestimated; limited substrate availability prevented us from fully evaluating the capacity through *in vitro* enzyme assays. Taken together, these unidentified protoberberine alkaloids constitute the ‘dark matter’ within the protoberberine repository. Future investigations employing high-resolution metabolomics could offer promising avenues to fully exploit the protoberberine metabolite network, ultimately providing a comprehensive understanding of the synthetic capabilities of *Coptis* species.

In this work, we also identified two unusual demethylases, Cc6DM2 and Cc6DM3, that belong to the 2-oxoglutarate-dependent dioxygenase family. In eukaryotic primary metabolism, members of this family can act as histone demethylases that dynamically regulate histone methylation [47]. This epigenetic regulation is associated with many crucial cellular processes and physiological activities [48]. Similarly, in protoberberine biosynthesis, Cc6DM2 and Cc6DM3 can remove the C-3 methyl group and methylenedioxy bridge of ring D, allowing demethylated metabolites to be redirected into different routes. We thus propose that Cc6DM2 and Cc6DM3 retain vestigial regulatory function from their common ancestor with histone demethylases. The protoberberine demethylases could collaborate with protoberberine *O*-methyltransferases and modulate the composition of the protoberberine repository through a methylation–demethylation tuning strategy. Previously, biosynthesis–regulation intersections have been observed in certain scenarios, where a natural product with DNA-binding activity can intercalate with the promoter region of a gene encoding a positive regulatory protein, thereby inhibiting the biosynthesis of the natural product itself [49, 50]. Similarly, by playing dual roles in protoberberine biosynthesis and the composition-tuning process, Cc6DM2 and Cc6DM3 might also constitute an intersection between biosynthesis and regulation. More examples that employ different paradigms of such intersection might be uncovered in the future to provide fresh perspectives on plant specialized metabolism [51].

In summary, the metabolite network could be a strategy employed not only by *C. chinensis* but also by various other plants to achieve chemical diversity and regulatory flexibility. It allows the production of a large metabolite repository with a minimal number of biosynthetic enzymes, an energy-efficient strategy that could facilitate the adaptation of the host plant. Future characterizations of the regulation of metabolite networks could provide us with a deeper understanding of their ecological roles and shed light on how plants use these networks to thrive in their environments.

## Materials and methods

### Plant materials and compounds

Three-year-old *C. chinensis* plants were collected from the Huanglian plantation base of Enshi city, Hubei province, China. The plants were transplanted to a greenhouse and grown at 25°C under a 16 h/8 h photoperiod. The plants had been adapted for 3 months before being used for RNA sequencing. Genes in this study were synthesized by GenScript Biotech Corporation (Nanjing, China). Protoberberine alkaloid standards were obtained from Wuhan ChemFaces Biochemical Co., Ltd. The compounds were dissolved in methanol to make stock solutions of 10 mM.

### RNA sequencing and data processing

RNA samples were prepared from rhizome, root and leaf tissue of *C. chinensis*, each with three biological replicates. For sequencing, a Qiagen RNeasy Kit was used for total RNA extraction, and quality was assessed with an Agilent 2100 Bioanalyzer. The sequencing was carried out on a HiSeq2000 sequencer (Illumina) in paired-end mode (PE100) by Shanghai Majorbio Bio-pharm Technology Co., Ltd. The quality of the sequenced reads (FASTQ files) was determined using FastQC. Trimmomatic was used to remove sequencing adapters and poor-quality reads [52]. The trimmed reads were assembled into transcripts, and the expression level of the transcripts was estimated using Trinity [53]. The putative ORFs were annotated using Trinotate (http://trinotate.github.io).

### Molecular cloning

Candidate genes in this study were synthesized by Genescript (China). All primers used in this study were synthesized by Tsingke Biotech Co. Ltd (Tsingke, China). CYP719 candidate genes amplified by PCR were inserted into BamHI/SalI-linearized pESC-HIS vector by Gibson assembly following the manufacturer’s protocols. The CcTHBO-encoding gene was amplified by PCR and inserted into BamHI/NotI-linearized pPIC3.5 k vector. The candidate *O*-methyltransferase and oxoglutarate-dependent dioxygenase-encoding genes were amplified by PCR and inserted into BamHI/HindIII-linearized pET28a expression vector. Clones were verified by diagnostic restriction digestion and sequencing. *Saccharomyces cerevisiae* transformation was performed as described in a previous study [54]. *Pichia pastoris* (GS115) transformation was performed by electroporation based on the manufacturer’s protocol (Invitrogen).

### Protein expression and purification

For the expression of CYP719 enzymes, yeast cells harboring the corresponding expression vector were grown in synthetic dextrose minimal medium (2% final concentration of dextrose) at 28°C, 220 rpm until OD_600_ reached 3.5. The yeast cells were washed three times and resuspended in synthetic galactose minimal medium (2% final concentration of galactose) and cultured at 28°C, 220 rpm for 48 h. After induction, yeast cells were collected and resuspended in TEK buffer (50 mM Tris–HCl, pH 7.4, 1 mM EDTA, 0.1 M KCl) and incubated at room temperature for 10 min. Cells were then resuspended in TESB buffer (50 mM Tris–HCl, pH 7.4, 1 mM EDTA, 0.1 M sorbitol) and lysed by vortexing with zirconia/silica disruption beads at 4°C. After centrifugation (16 000 *g*, 40 min, 4°C), the supernatant was supplemented with PEG 3350 (final concentration of 0.1 g/ml) and NaCl (final concentration of 0.15 M). The mixture was agitated on ice for 30 min before centrifugation at 30 000 *g* for 40 min. The resulting microsome fraction was homogenized in TEG buffer (50 mM Tris–HCl, pH 7.4, 1 mM EDTA, 20% glycerol) using a Dounce homogenizer. The protein concentration of the extracted microsomes was determined using Bradford’s assay. Microsomes were aliquoted, flash-frozen and stored at −80°C.

For the expression of CcTHBO, the recombinant *P. pastoris* strain GS115 harboring the expression vector was grown in BMGY medium for 18–20 h at 28°C, 250 rpm until OD_600_ reached 2–6. The cells were washed three times and resuspended in BMMY medium for incubation at 28°C, 250 rpm for 96 h. The culture was supplemented with 0.5% (v/v) methanol every 12 h. Cell pellets were collected by centrifugation (2000 *g*, 5 min) and disrupted with a French press (35 000–40 000 psi kpsi, 10 min) in lysis buffer (50 mM Tris–HCl, pH 7.5, 300 mM NaCl, 10 mM imidazole, 10% glycerol). Lysate was clarified by centrifugation (10 000 *g*, 40 min) and affinity-purified with a HisTrap protein purification column. Target-protein-containing fractions, as determined by SDS–PAGE, were pooled and dialyzed overnight at 4°C against protein preservation buffer (in 20 mM Tris, pH 7.5, 100 mM NaCl, 20% glycerol). The resulting protein solution was concentrated by Amicon Ultra-15 Centrifugal Filters (Millipore), flash-frozen and preserved at −80°C.

For the expression of *O*-methyltransferases and oxoglutarate-dependent oxygenases, *E. coli* strain BL21(DE3) harboring the expression vector was grown in Luria–Bertani (LB) broth until OD_600_ reached 0.6. Protein overproduction was induced with 0.2 mM isopropyl-β-d-thiogalactoside and incubated at 16°C, 250 rpm for 20 h. Cells were collected by centrifugation (7000 *g*, 20 min) and lysed by ultrasonication (200 Hz, 40 min). The protein purification procedure was the same as that for CcTHBO purification.

### *In vitro* enzyme assays

The *in vitro* activity of CYP719 enzymes was assayed in reaction volumes of 100 μl, containing 100 mM Tris–HCl buffer (pH 7.5), NADPH Regeneration System (Promega) according to the manufacturer’s instructions, 0.2 mM substrate, and 10 μg of microsome protein. The reaction systems were incubated at 30°C for 6 h. Microsomes extracted from the *S. cerevisiae* strain WAT11 strain harboring empty pESC-HIS vector were assayed as negative control.

CcTHBO enzyme assays were performed in a 100-μl reaction system containing 100 mM K_2_HPO_4_/KH_2_PO_4_ buffer (pH 8.0), 0.2 mM substrate, and 20 μM recombinant protein. The reaction was carried out in a foil-wrapped 1.5-ml Eppendorf tube to avoid light. The reactions were carried out at 30°C for 4 h. Boiled CcTHBO was used as the negative control.

For the assays of *O*-methyltransferases, the 100-μl reaction system contained 100 mM HEPES buffer system (pH 7.5), 1 mM ascorbic acid, 10 mM *S*-adenosyl methionine (SAM), 0.5 mM substrate, and 10 μM purified enzyme. The reaction was carried out at 30°C for 2 h. Boiled CcOMT1 was used as the negative control.

For the assays of oxoglutarate-dependent oxygenases, the 100-μl reaction system contained 100 mM Tris–HCl buffer system (pH 7.5), 10 mM ascorbic acid, 1 mM α-ketoglutarate, 0.5 mM FeSO_4_, 14 mM β-mercaptoethanol, 0.5 mM substrate, and 10 μM purified enzyme. The reaction mixtures were incubated at 30°C for 4 h. Boiled protein was used as the negative control.

### Protein structure modeling and substrate docking

The structures of CcCYP719A1 and CcCYP719A2 were modeled using AlphaFold, which was available at Google Colab (Google, USA) [55]. Ligand-docking calculations (cheilanthifoline, scoulerine, and tetrahydrocolumbamine) were performed using AutoDock Vina (version 1.5.7). The molecular representations were displayed and rendered with PyMOL 2.5.0.

### Sequence evolution analysis

Nucleotide sequences of CYP719 family proteins, along with *CcCYP719A1–3*, were aligned based on codon sequences using MUSCLE implemented in MEGA X with default parameters [56]. The resulting alignment was used to construct a maximum likelihood tree, whose bootstrap percentages were calculated based on 1000 replications. Estimation of the lineage-specific rate of the non-synonymous/synonymous (*d*_N_*/d*_S_) rate ratio (ω) among multiple lineages was conducted based on the free-ratio branch model implemented in CODEML from the PAML package [23]. To detect if positive selection had acted on the branch containing CYP719A19, CcCYP719A2, and CcCYP719A3, a branch-site analysis under ‘branch-site model A’ was performed (model = 2, NSsites = 2), treating this branch as the foreground lineage while all other branches were treated as background branches [24].

### HPLC analysis

Typically, 100 μl ice-cold methanol was used for quenching the reaction. The mixture was then centrifuged (10 000 *g*, 4°C, 20 min) to remove protein precipitate and filtered before HPLC analysis. Ten microliters of freshly prepared sample was subjected to reverse-phase HPLC analysis, using a Shimazu LC-2030 Plus HPLC system. Separation was carried out on a Thermo Fisher Hypersil BDS C18 column using water (0.1% formic acid, solvent A) and acetonitrile (0.1% formic acid, solvent B). The column was eluted with the following gradient: 10–50% B for 5 min, 0–90% B for 20 min, 90–95% B for 1 min, 100% B for 4 min, 100–10% B for 2 min, and 10% B for 10 min, flow rate at 0.8 ml/min. The column was maintained at 35°C and absorption was observed at 278 nm.

### LC–MS analysis

Typically, 2 μl of the filtered sample was used for LC–MS analysis. The reaction products in this study were identified by comparing their *m*/*z* value and MS^2^ fragmentation pattern with standard compounds. The LC separation was carried out using a Thermo Fisher UltiMate 3000 UHPLC system, employing an Agilent Zorbax 300SB-C8 column. Separation was achieved through a gradient elution process using water (0.1% formic acid, solvent A) and acetonitrile (0.1% formic acid, solvent B): 10–50% B for 5 min, 50–90% B for 20 min, 90–100% B for 1 min, 100% B for 9 min, 100–10% B for 1 min, and 10% B for 10 min, flow rate at 0.3 ml/min. For LC–MS/MS analysis, a Thermo Scientific LTQ XL Orbitrap mass spectrometer was utilized, operating in the positive ion mode. The specific MS analysis settings were as follows: 45 V capillary voltage, 45°C capillary temperature, auxiliary gas flow rate 10 arbitrary units, sheath gas flow rate 40 arbitrary units, 3.5 kV spray voltage, and 50–1000 amu mass range (maximum resolution 30 000).

## Acknowledgements

This work was supported by Guangxi Science and Technology Base and Talent Special Project (AD23026030) to Y.Y. and B.W. This work was also supported by grants to Y.Y. from the National Key Research and Development Program of China (2018YFA0900400), the Guiding Funds of Central Government for Supporting the Development of the Local Science and Technology (ZY23055032), and the National Natural Science Foundation of China (22277095). L.W. is funded by the International Postdoctoral Exchange Fellowship Program, China Postdoctoral Council (YJ20200331) and the Fundamental Research Funds for the Central Universities (2042021kf0074).

## Author contributions

Y.Y. and Z.D. planned and designed the research. L.W. and B.Z. performed the experiments. L.W., B.Z., and B.W. analyzed the data; and Y.Y. and L.W. wrote the manuscript.

## Data availability statement

The data that support the findings of this study are openly available in the National Center for Biotechnology Information (NCBI) at https://www.ncbi.nlm.nih.gov, accession number OP168911-OP168915, OR670424- OR670426.

## Conflict of interests

The authors declare no competing interests.

## Supplementary information

Supplementary data is available at *Horticulture Research* online.

## Supplementary Material

Web_Material_uhad259
