# Supplementary material for: A biosynthetic network for protoberberine production in Coptis chinensis
Source: Hortic Res. 2023 Dec 13;11(1):uhad259. doi: 10.1093/hr/uhad259 (PMC10812381; doi:10.1093/hr/uhad259)
Supplement: Web_Material_uhad259 [file web_material_uhad259.zip › MR_SI.docx]

**Supplemental information**

A biosynthetic network for protoberberine production in *Coptis chinensis.*

Linrui Wu^1^, Binxin Zhao^1^, Zixin Deng^1^, Bin Wang^2^, Yi Yu^*1,2^

1. Department of Gastroenterology, Zhongnan Hospital of Wuhan University, Hubei Clinical Center and Key Laboratory of Intestinal and Colorectal Disease, School of Pharmaceutical Sciences, Wuhan University, 185 East Lake Road, Wuhan, P. R. China.

2. National Engineering Research Center for Non-food Biorefinery, Guangxi Academy of Sciences, Nanning, P. R. China.

*Correspondence: E-mail: yu_yi@whu.edu.cn; Tel: +86(27)68752491


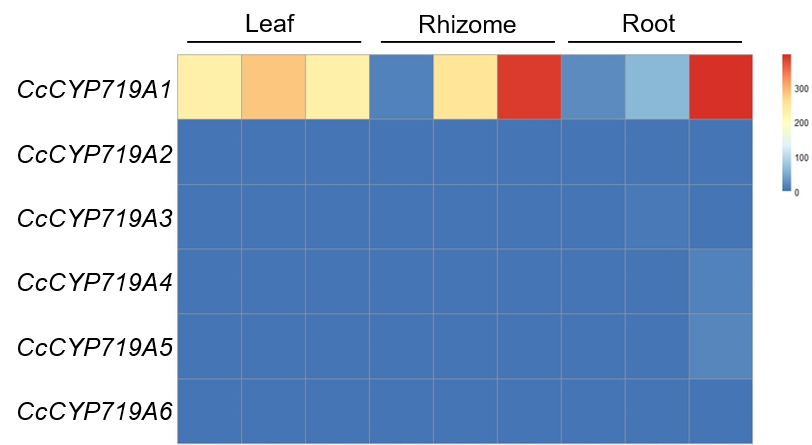


Figure S1. The heatmap representing the expression level of six candidate CYP719 encoding transcripts identified from *C. chinensis*.


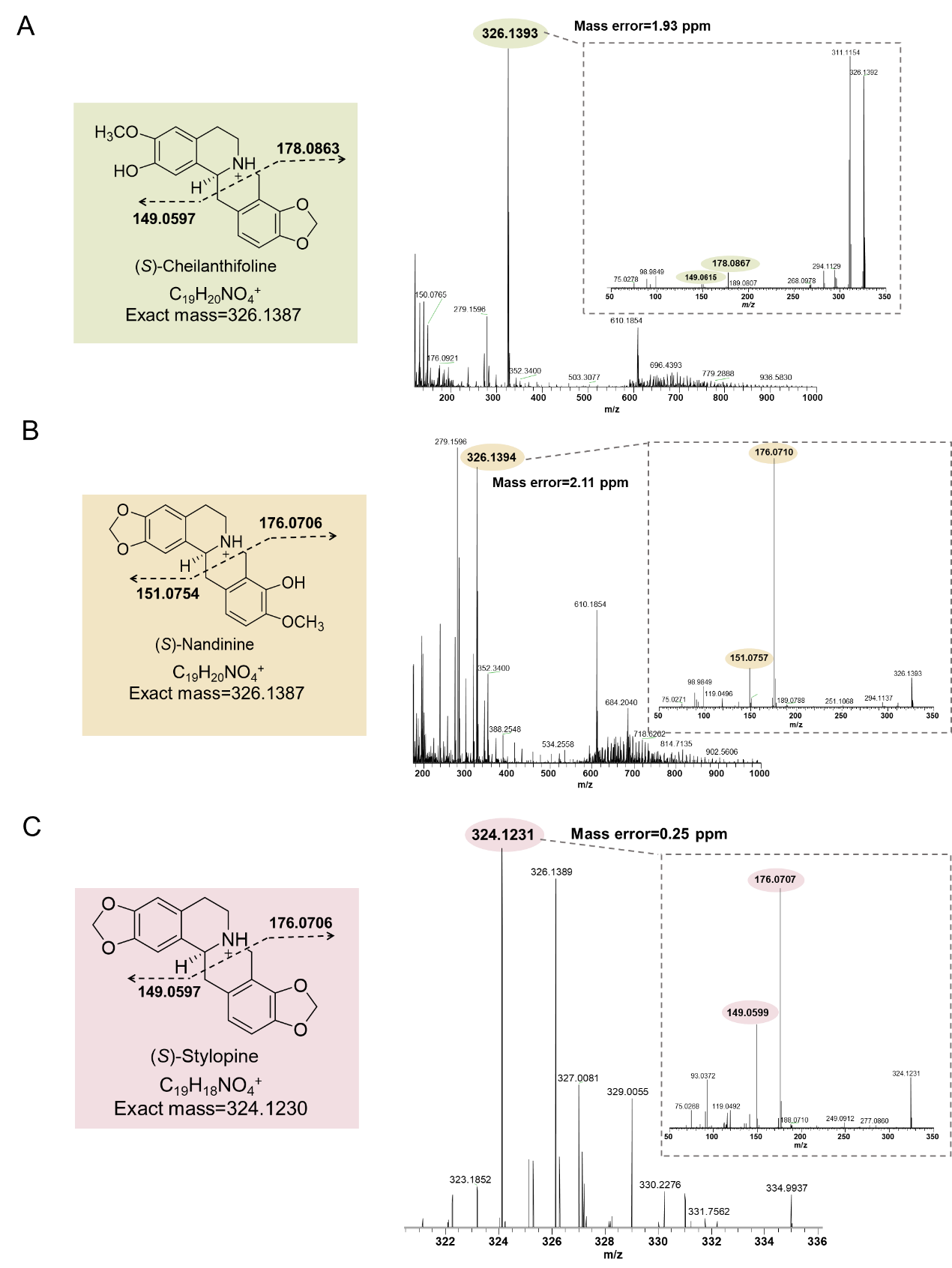


Figure S2. The MS^2^ spectrum of the reaction products from CYP719 enzyme assays.

(A) The MS^2^ fragmentation of (*S*)-cheilanthifoline.

(B) The MS^2^ fragmentation of (*S*)-nandinine

(C) The MS^2^ fragmentation of (*S*)-stylopine


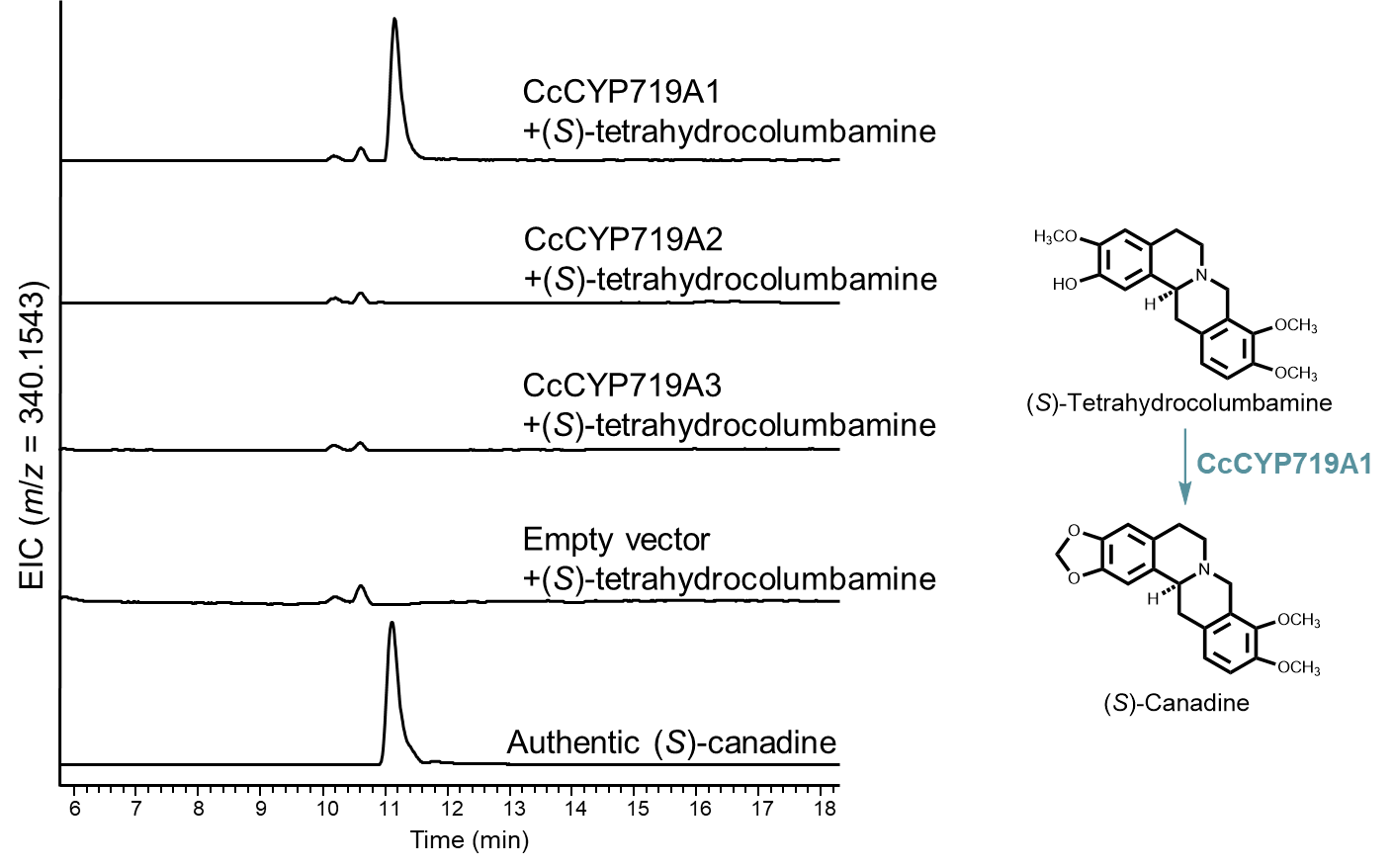


Figure S3. Extracted ion chromatograms of biochemical assay of CYP719 candidates using (*S*)-tetrahydrocolumbamine as substrate, according to the *m*/*z* values of the product (*S*)-canadine.


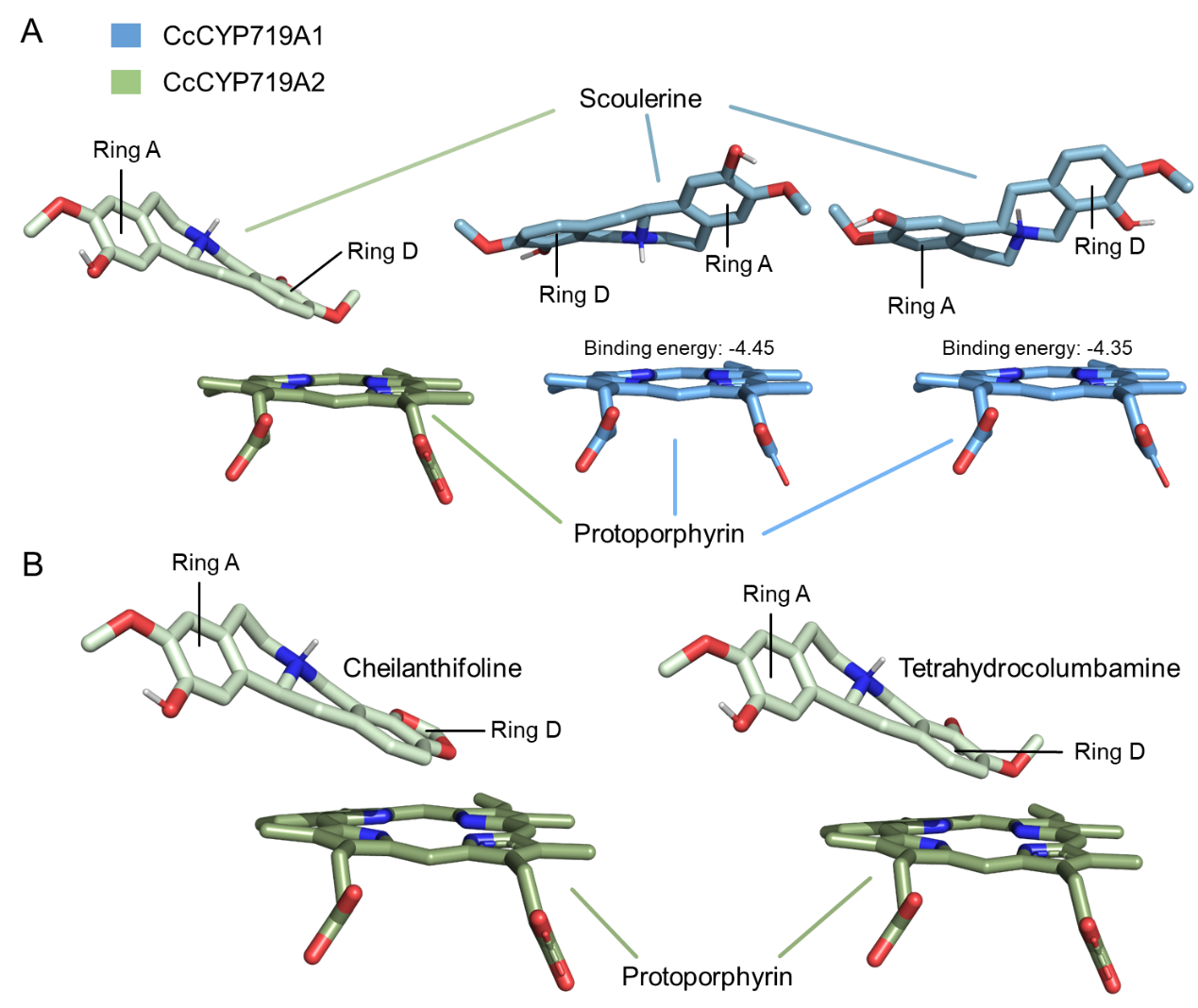


Figure S4. The structural model of ligand binding in the active site of CcCYP719A1 and CcCYP719A2.

(A) The orientation of (*S*)-scoulerine differs in CcCYP719A1 (blue) and CcCYP719A2 (green). (*S*)-Scoulerine adopts a slanted conformation in CcCYP719A2, and thereby more restricted in its catalytic pocket. Whereas in CcCYP719A1, (*S*)-scoulerine is positioned in parallel of the protoporphyrin plane, allowing it to enter the active site in two possible orientations.

(B) Modelling of (*S*)-cheilanthifoline and (*S*)-tetrahydrocolumbamine in the active site of CcCYP719A2. These substrates adopted a similar slanted conformation as (*S*)-scoulerine that the ring-D is oriented towards the catalytic heme.


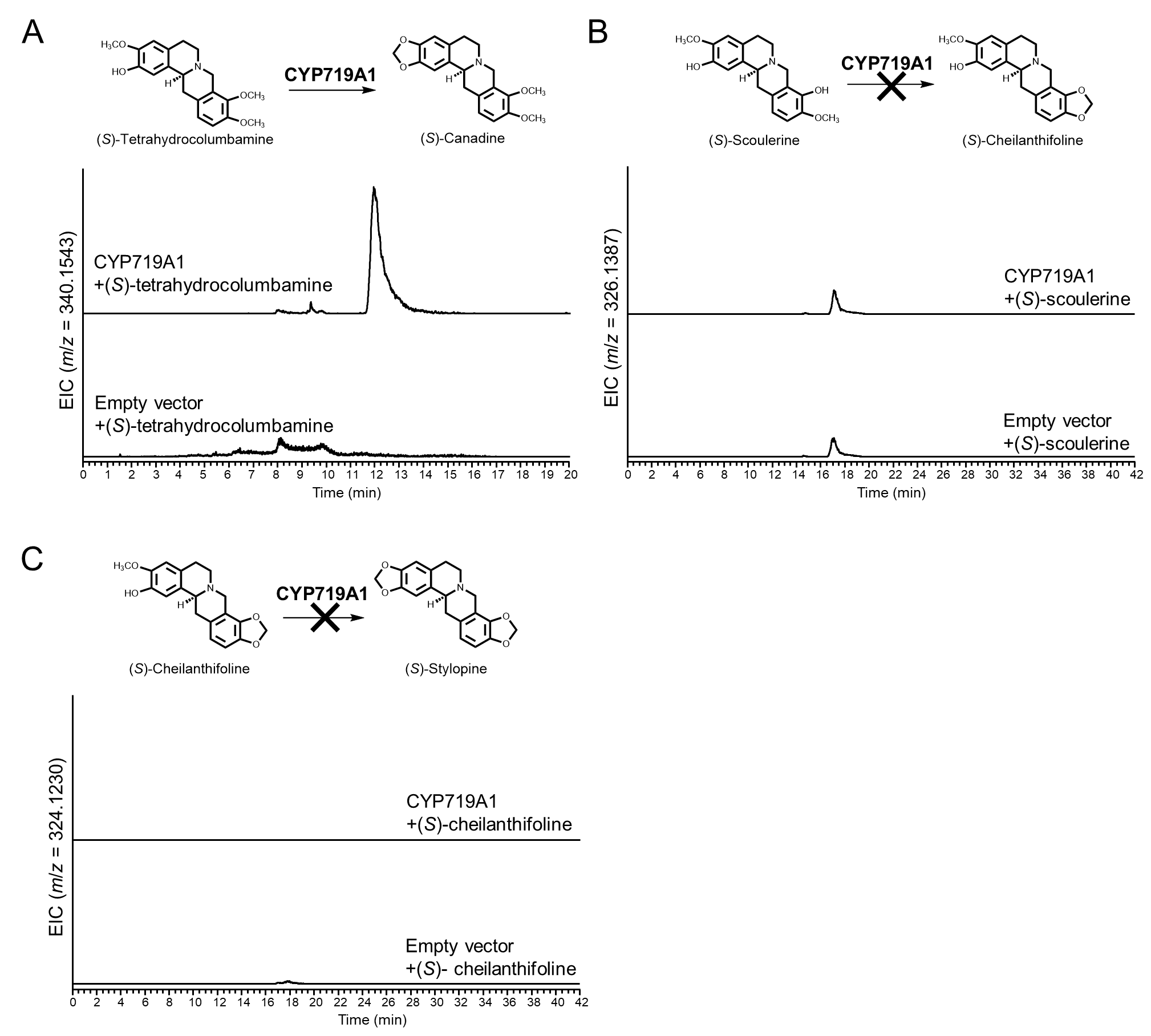


Figure S5. The functional characterization of CYP719A1.

(A) Extracted ion chromatograms according to the theoretical *m*/*z* value of (*S*)-canadine, showing the enzyme assays of CYP719A1 using (*S*)-tetrahydrocolumbamine as substrate.

(B) Extracted ion chromatograms according to the theoretical *m*/*z* value of (*S*)-cheilanthifoline, showing the enzyme assays of CYP719A1 using (*S*)-scoulerine as substrate.

(C) Extracted ion chromatograms according to the theoretical *m*/*z* value of (*S*)-stylopine, showing the enzyme assays of CYP719A1 using (*S*)-cheilanthifoline as substrate.


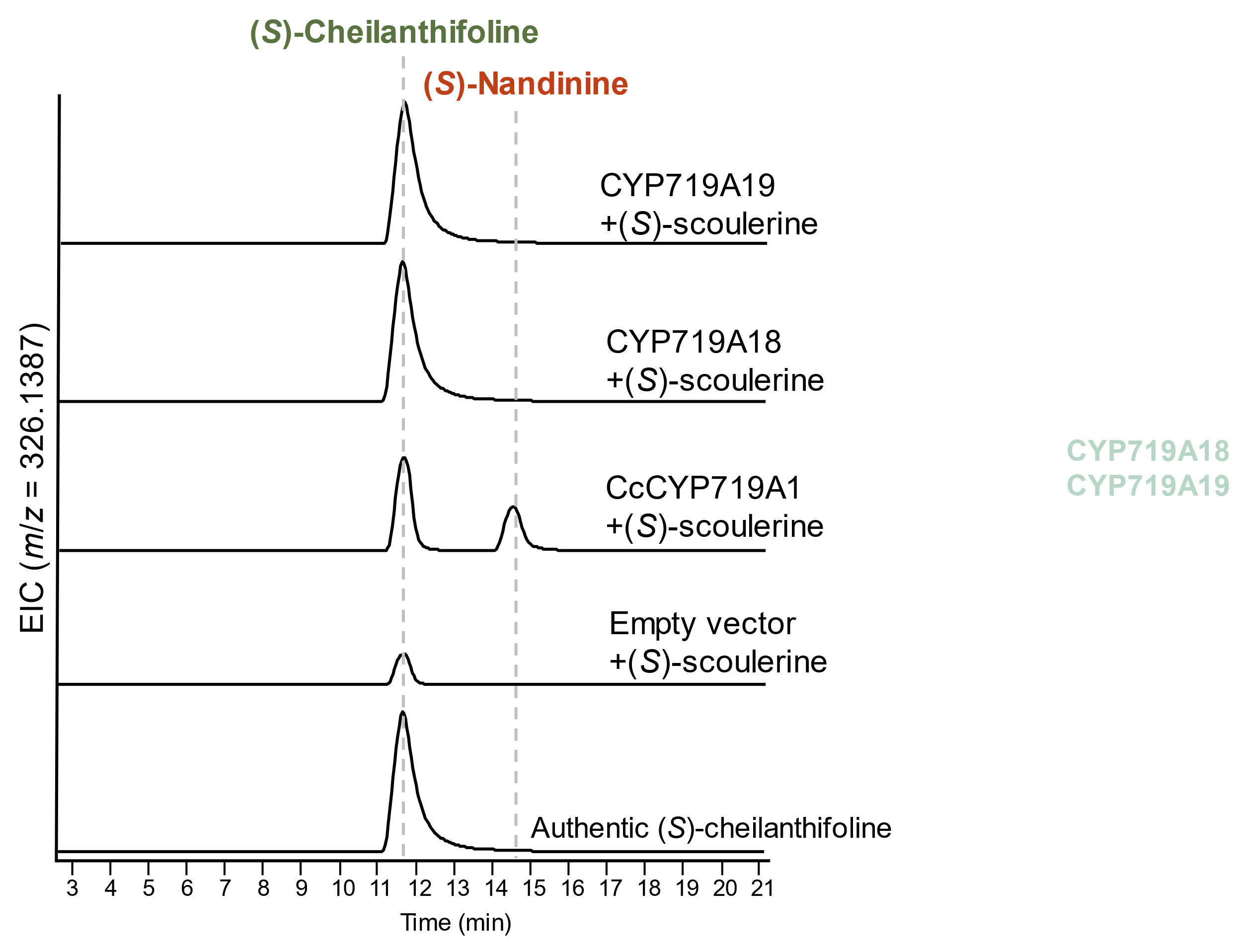


Figure S6. Extracted ion chromatograms according to the theoretical *m*/*z* value of (*S*)-cheilanthifoline, showing the enzyme assays of CYP719A18 and CYP719A19. CcCYP719A1 was used as positive control, microsomes extracted from WAT11 harbouring the pESC-HIS empty vector was used as negative control.


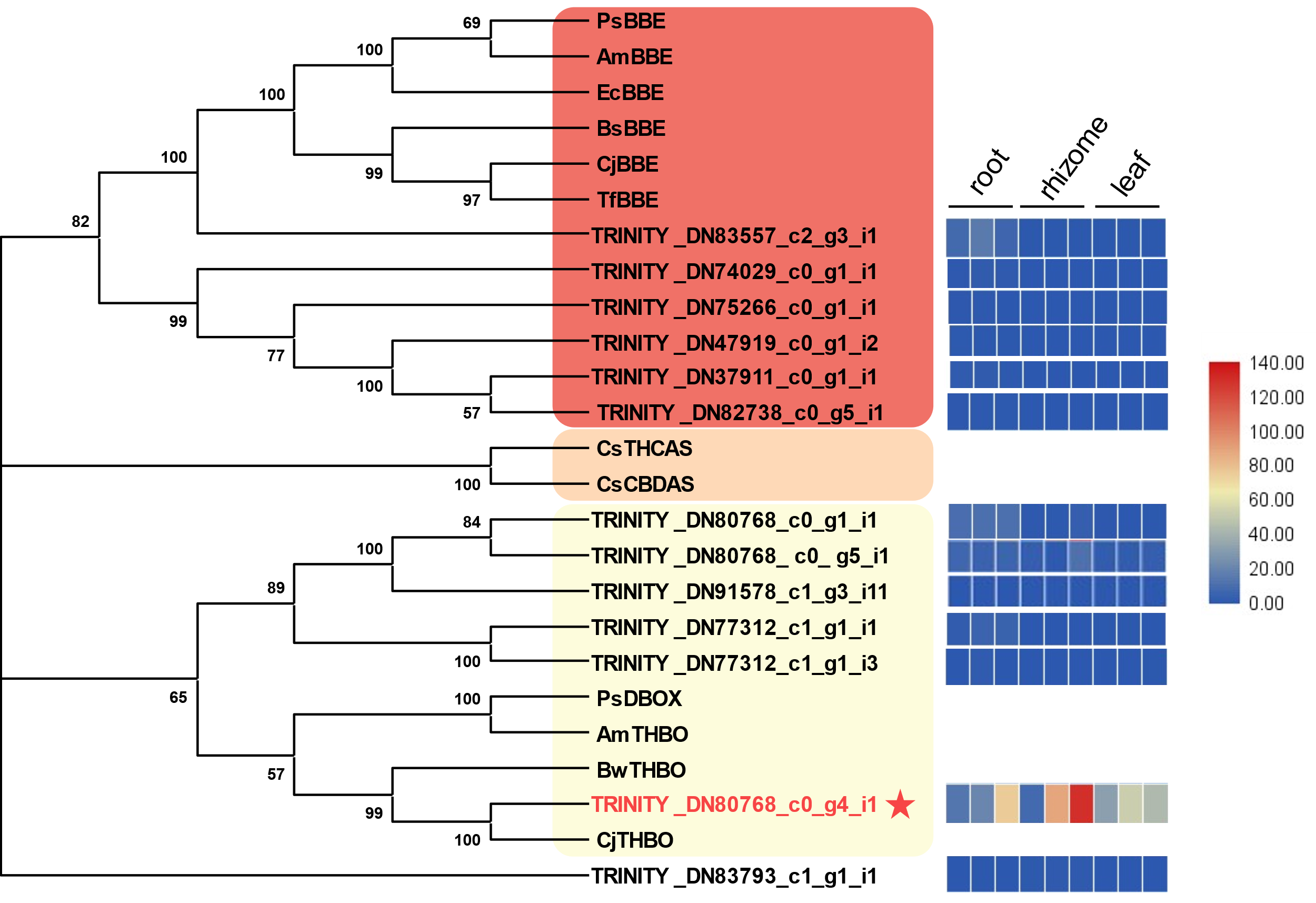


Figure S7. The phylogenetic analysis for the identification of THBO candidate from *C. chinensis*. The heatmap on the right panel shows the expression level of the identified candidate THBO transcripts from BLASTP and Pfam domain search. The CcTHBO characterized in this study is marked with red asterisk.


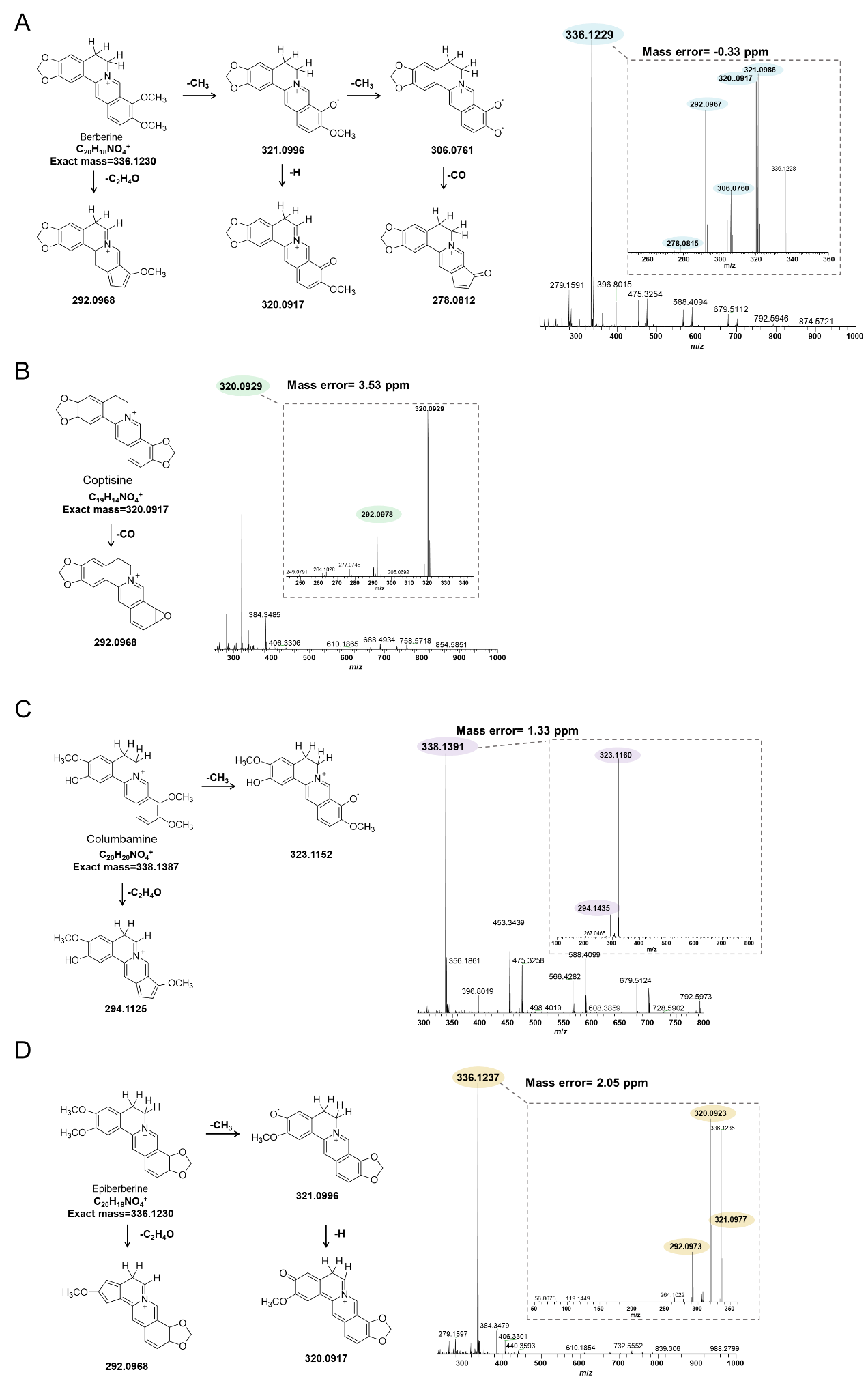


Figure S8. The MS^2^ analysis of products from CcTHBO in vitro assay.

(A) The MS^2^ fragmentation of berberine.

(B) The MS^2^ fragmentation of coptisine.

(C) The MS^2^ fragmentation of columbamine.

(D) The MS^2^ fragmentation of epiberberine.


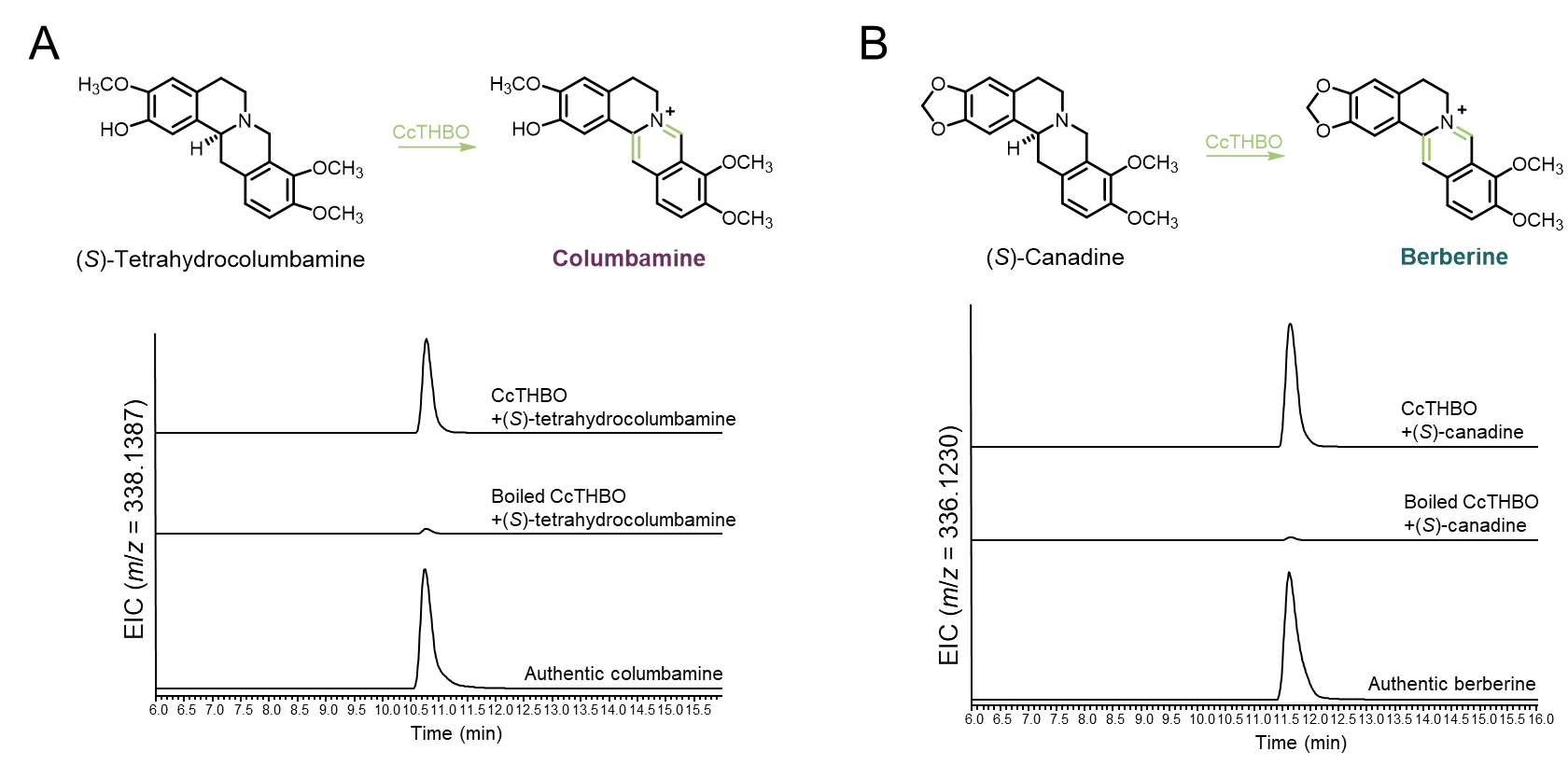


Figure S9. The enzyme assays of CcTHBO using (*S*)-canadine and (*S*)-tetrahydrocolumbamine as substrates.

(A) The production of columbamine from (*S*)-tetrahydrocolumbamine.

(B) The production of berberine from (*S*)-canadine.


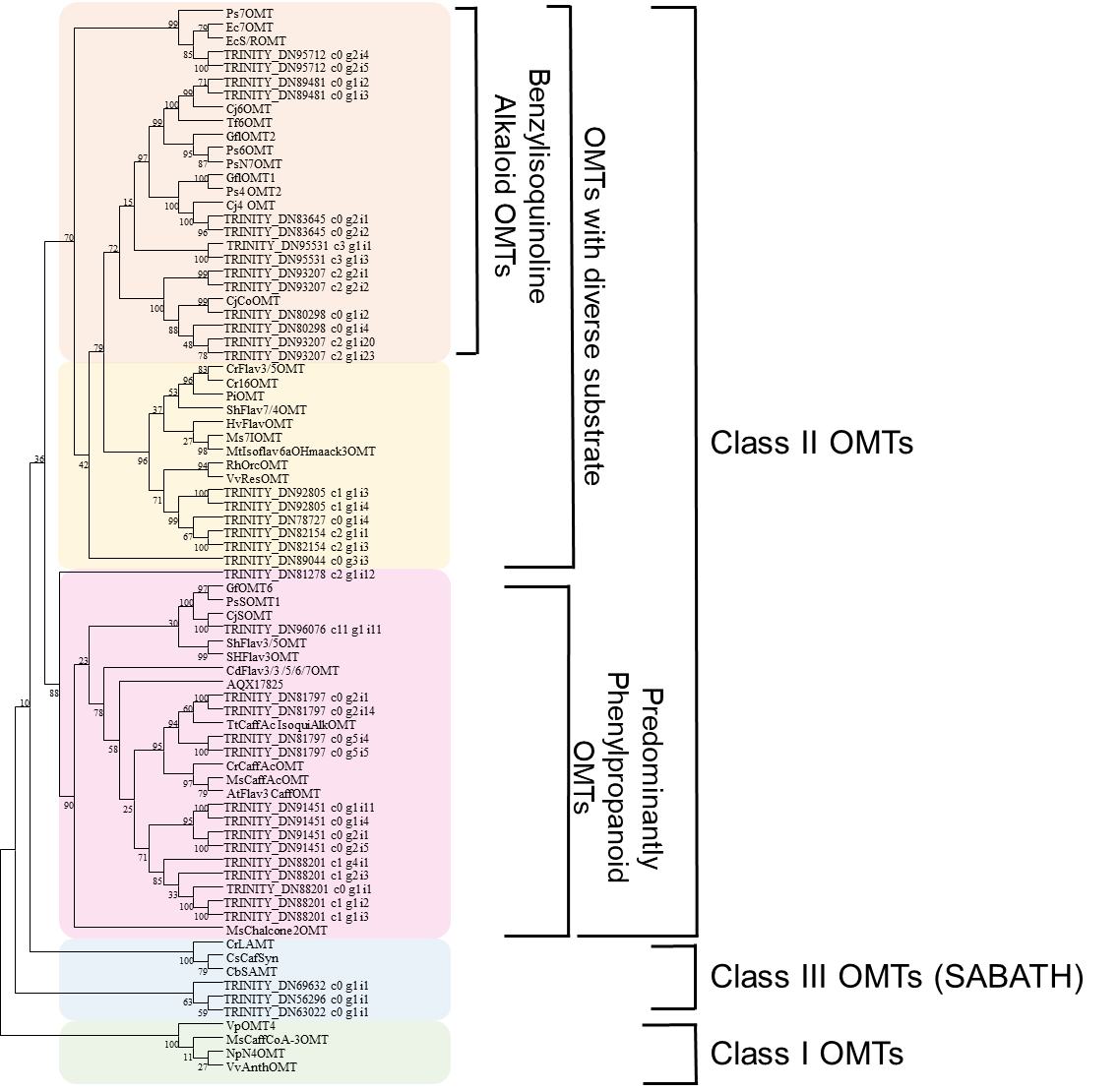


Figure S10. The phylogenetic analysis of OMT candidates from *C. chinensis* with OMTs from three major families. Among the 38 candidates, 14 were clustered with OMTs that has been characterized to be responsible for BIA biosynthesis.


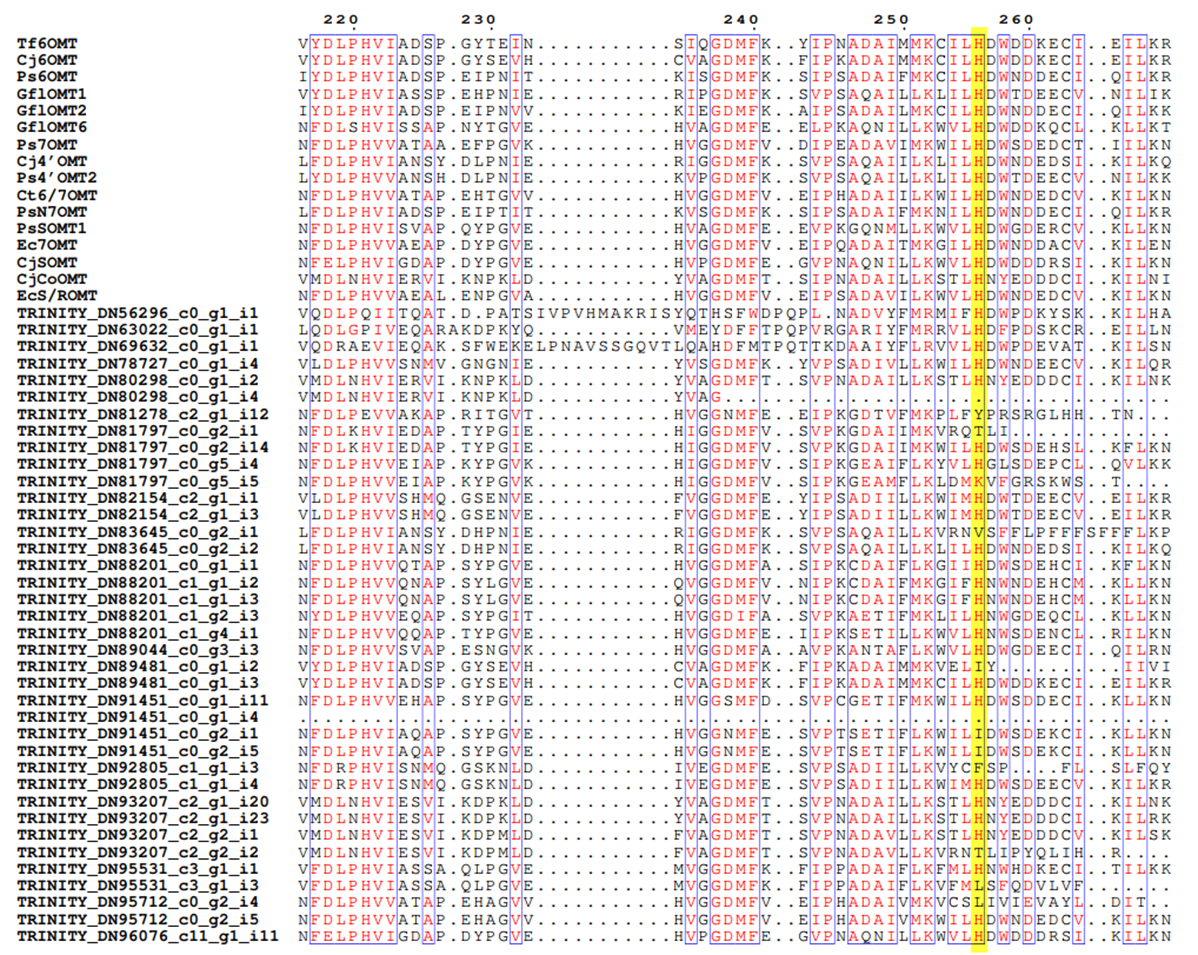


Figure S11. The multiple sequence alignment of OMT candidates from *C. chinensis* with characterized OMTs responsible for BIA biosynthesis. Transcripts without the critical His256 that forms hydrogen bond with the hydroxy group on the substrate are eliminated from further analysis[[1](#_ENREF_1)].


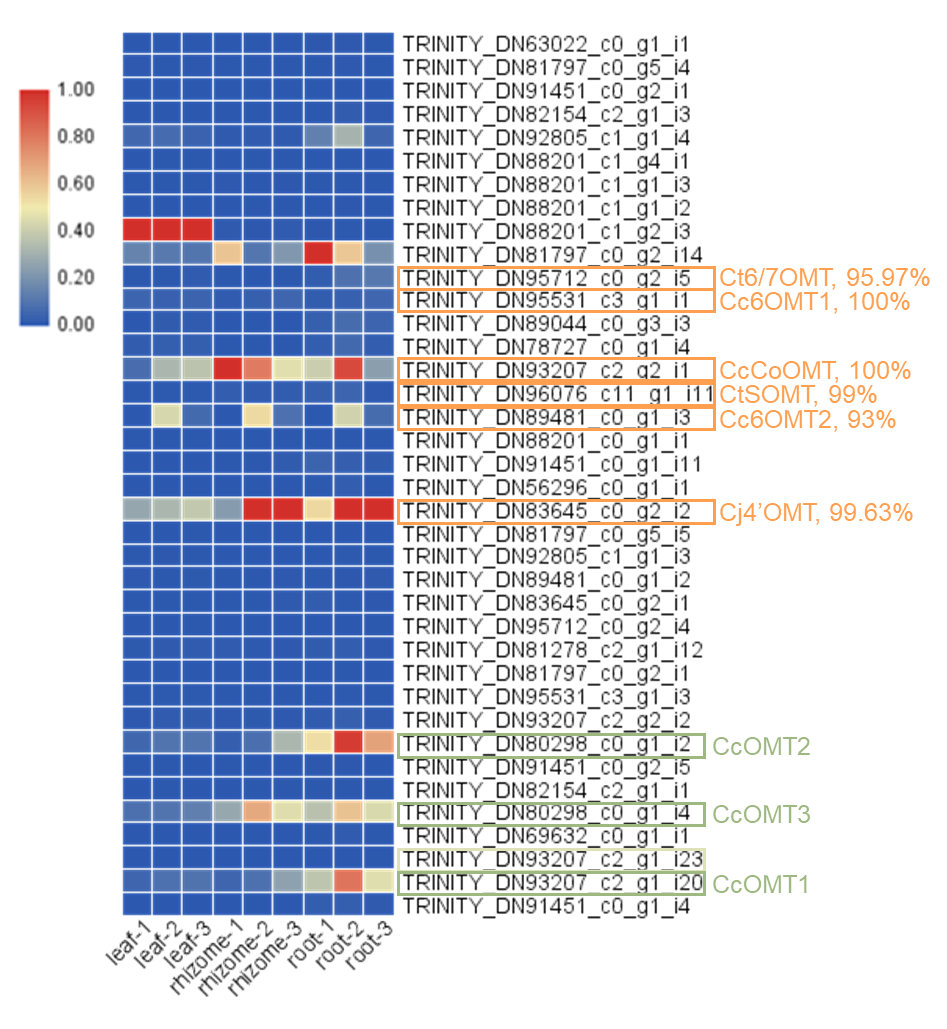


Figure S12. The identification of candidate OMT transcripts for biochemical characterisation. Transcripts whose encoded protein shares >90% amino acid identity with characterized OMTs responsible for BIA biosynthesis are indicated in orange font. Transcripts with minimal expression level across all samples were eliminated from further analysis. The selected three transcripts were thereby referred as CcOMT1-3.


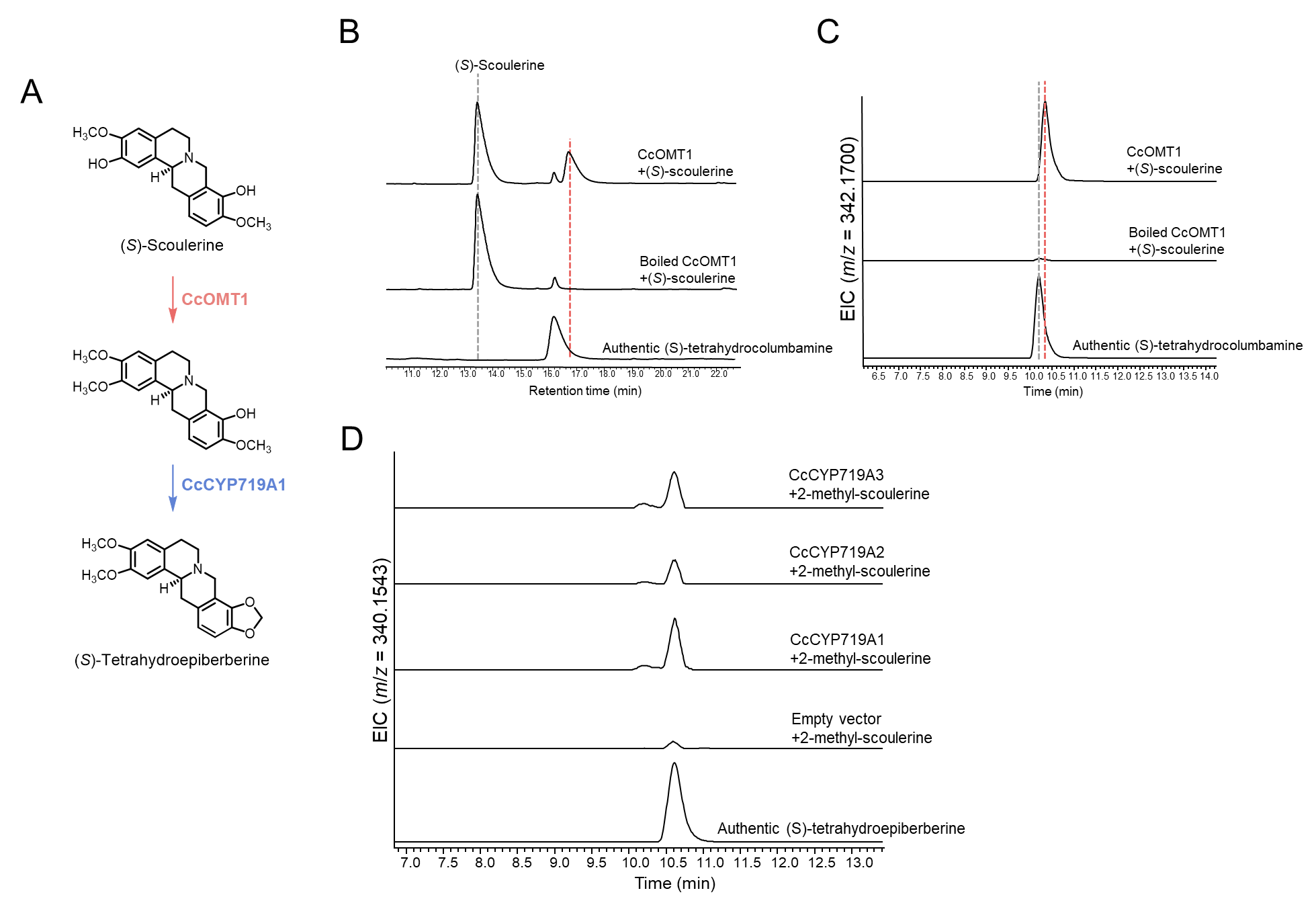


Figure S13. The alternative route for the transformation from (*S*)-scoulerine to (*S*)-tetrahydroepiberberine.

(A) The proposed stepwise methylation and methylenedioxy bridge formation of (*S*)-scoulerine to produce (*S*)-tetrahydroepiberberine.

(B) HPLC analysis of the CcOMT1 enzyme assay using (*S*)-scoulerine as substrate. Boiled protein was used for negative control. The methylation product elutes earlier than authentic (*S*)-tetrahydrocolumbamine, indicating that the methylation occurs at C-2, rather than C-9.

(C) Extracted ion chromatograms of CcOMT1 enzyme assays according to the theoretical *m*/*z* values of methylated (*S*)-scoulerine.

(D) Extracted ion chromatograms of CYP719 enzyme assay using methylated scoulerine generated by CcOMT1 as substrate, according to the theoretical *m*/*z* values of (*S*)-tetrahydroepiberberine. Microsomes extracted from WAT11 harbouring the pESC-HIS empty vector was used for negative control.

Figure S14. The common precursor pathway for protoberberine alkaloids. From the condensation of dopamine and 4-hydroxylphenylacetaldehyde that generates (*S*)-norcoclaurine, to the production of (*S*)-scoulerine by berberine bridge enzyme[[2](#_ENREF_2)].


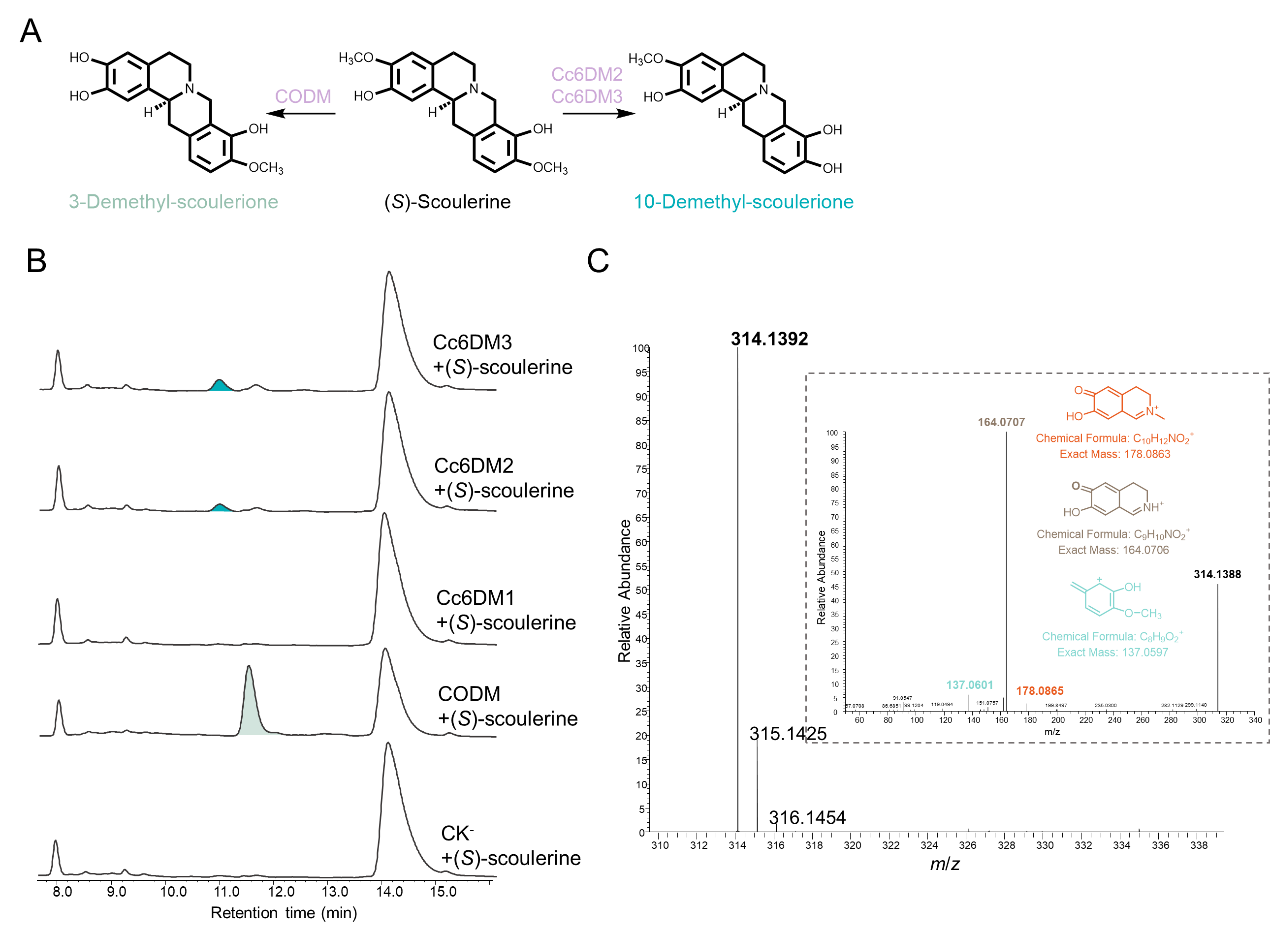


Figure S15. The demethylation of (*S*)-scoulerine.

(A) The proposed demethylation reactions of (*S*)-scoulerine catalyzed by CODM and Cc6DM2/Cc6DM3, respectively.

(B) HPLC analysis of the candidate demethylase enzyme assays using (*S*)-scoulerine as substrate.

(C) The MS^2^ spectrum of the demethyl-scoulerine generated by CODM.


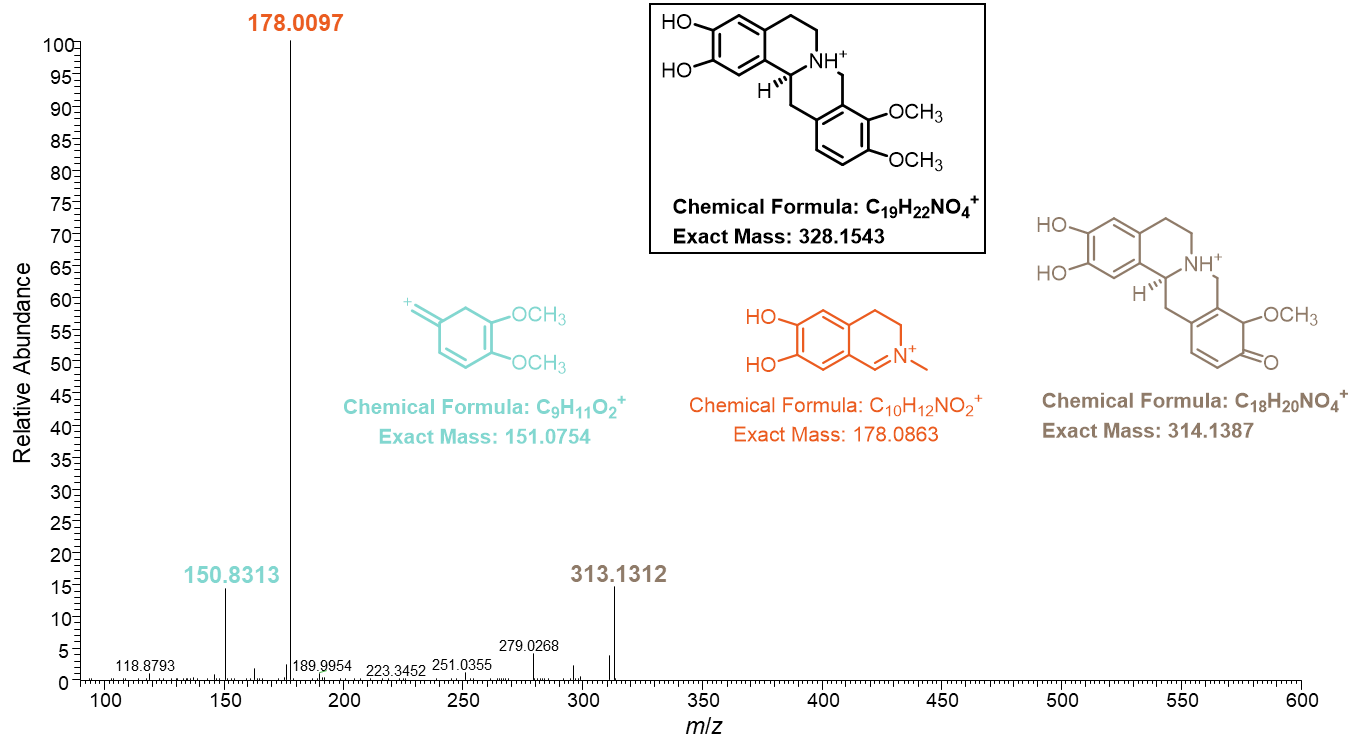


Figure S16. The MS^2^ analysis of the demethylation product of (*S*)-tetrahydrocolumbamine catalyzed by Cc6DM2/Cc6DM3.


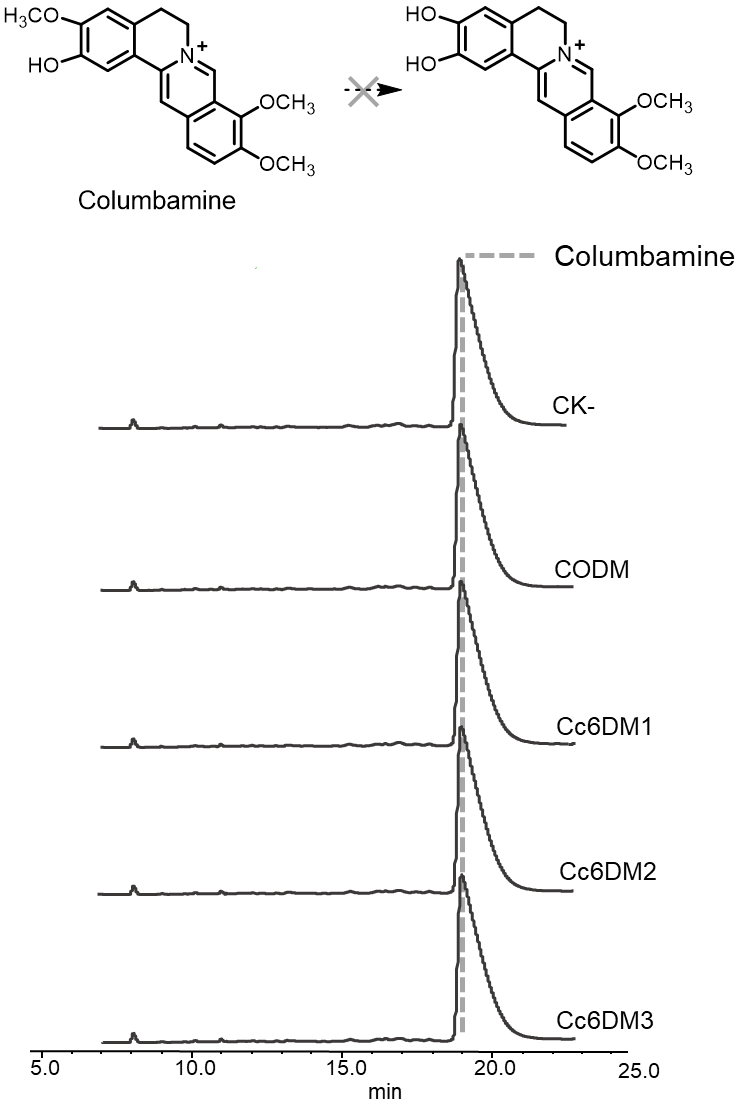


Figure S17. HPLC analysis of enzyme assays of the demethylase candidates using columbamine as substrate.


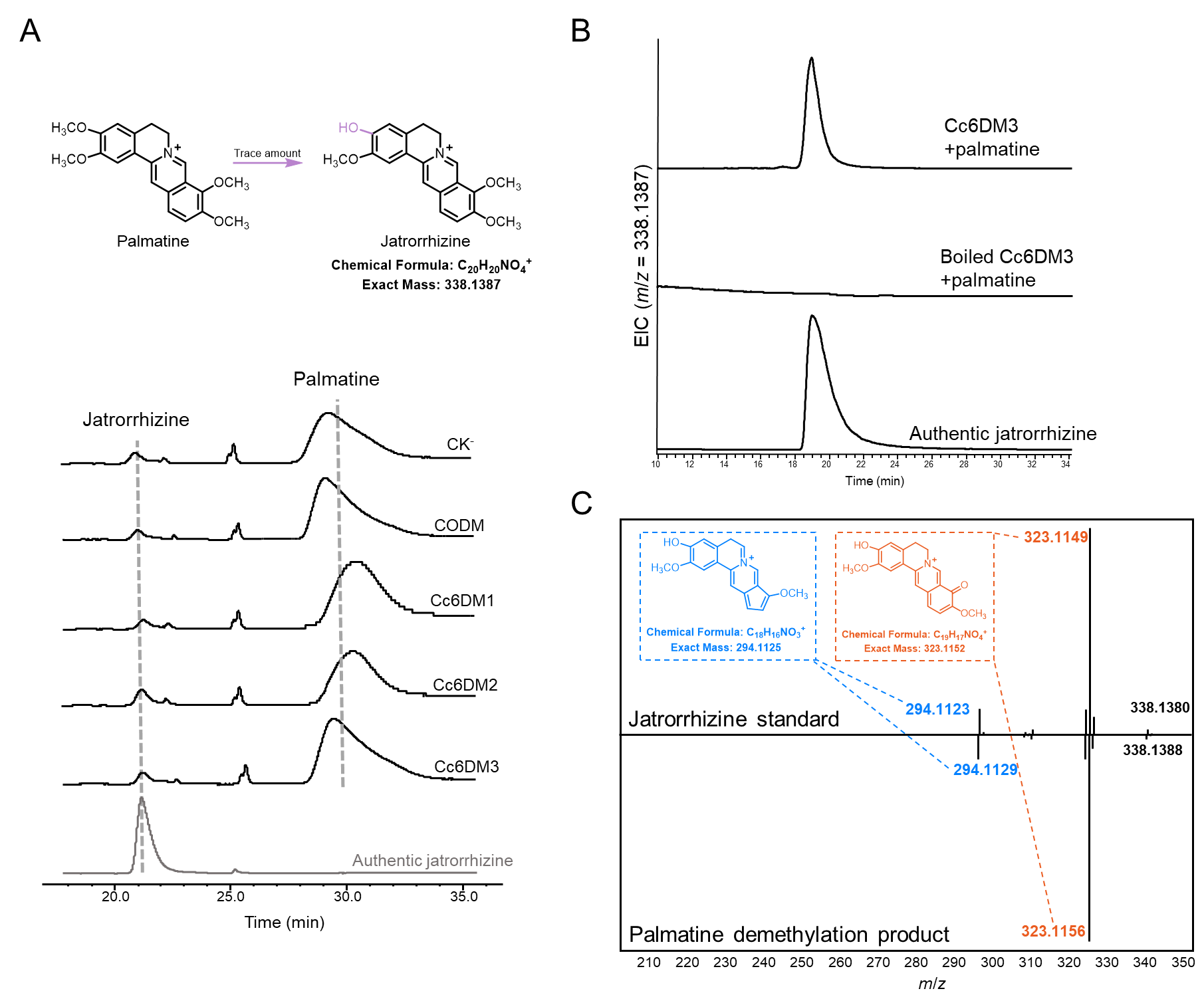


Figure S18. The production of jatrorrhizine from demethylation of palmatine.

(A) The schematic representation of the demethylation of palmatine.

(B) Extracted ion chromatograms of Cc6DM3 enzyme assay using palmatine as substrate, according to the theoretical *m*/*z* values of jatrorrhizine, boiled Cc6DM3 was used for negative control.

(C) The MS^2^ spectrum of Cc6DM3 enzyme assay product, the spectrum of authentic jatrorrhizine was used for comparison.


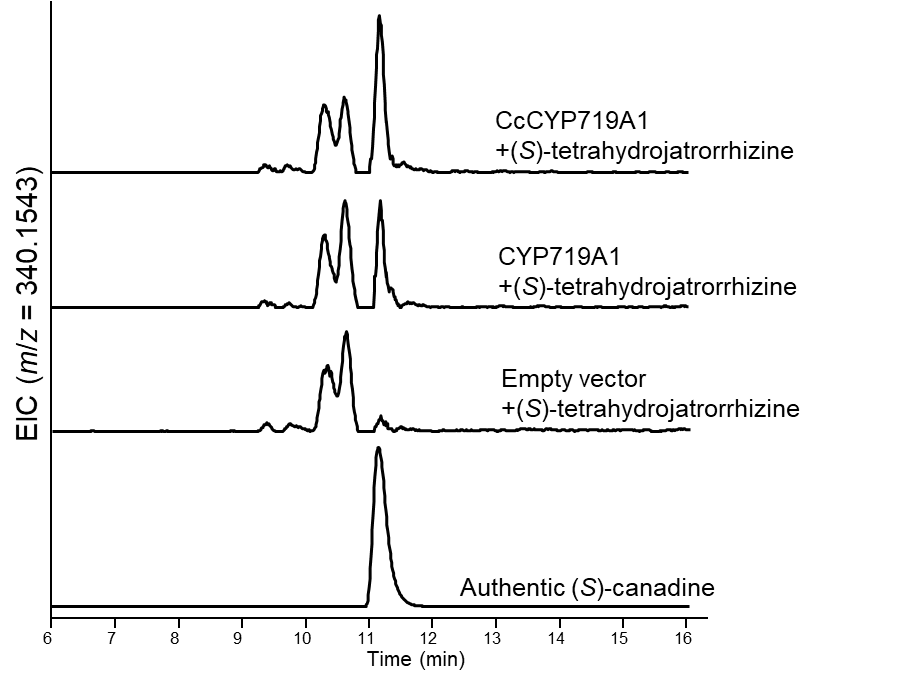


Figure S19. Extracted ion chromatograms of CcCYP719A1 and CYP719A1 enzyme assays using (*S*)-tetrahydrojatrorrhizine as substrate, according to the theoretical *m*/*z* value of (*S*)-canadine.


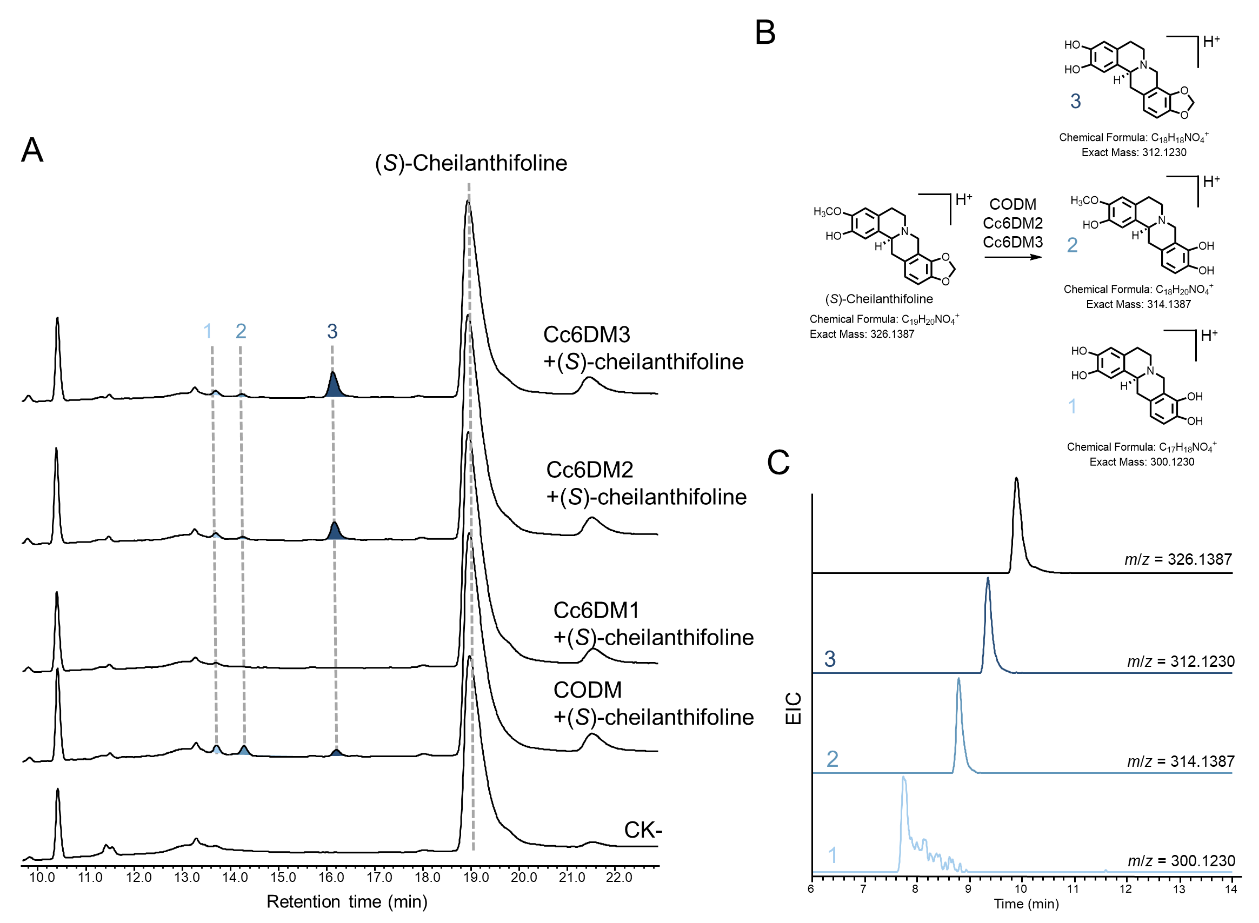


Figure S20. The *O*-demethylation and *O*,*O*-demethylenation of (*S*)-cheilanthifoline.

(A) HPLC analysis of the enzyme assays of demethylase candidates using (*S*)-cheilanthifoline as substrate. CODM was used as control.

(B) The schematic representation of proposed *O*-demethylation and *O*,*O*-demethylenation of (*S*)-cheilanthifoline.

(C) The extracted ion chromatograms according to the *m*/*z* values of the expected *O*-demethylation and *O*,*O*-demethylenation products from the Cc6DM3 enzyme assay.


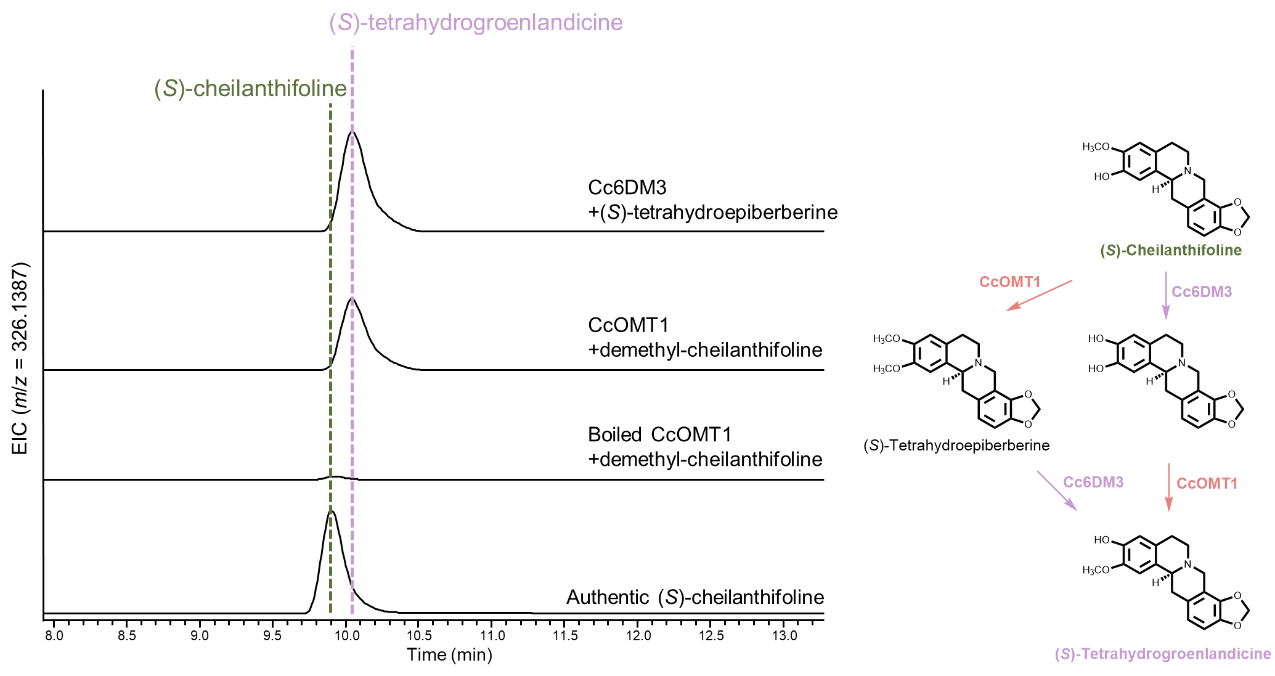


Figure S21. The extracted ion chromatograms according to the *m*/*z* value of demethylated (*S*)-tetrahydroepiberberine, showing the enzyme assay of CcOMT1 using demethylated (*S*)-cheilanthifoline (generated by Cc6DM3 from (*S*)-cheilanthifoline) as substrate. The enzyme assay of Cc6DM3 using (*S*)-tetrahydroepiberberine as substrate was used for comparison, indicating that the two assays generated the same product, which is different from (*S*)-cheilanthifoline.


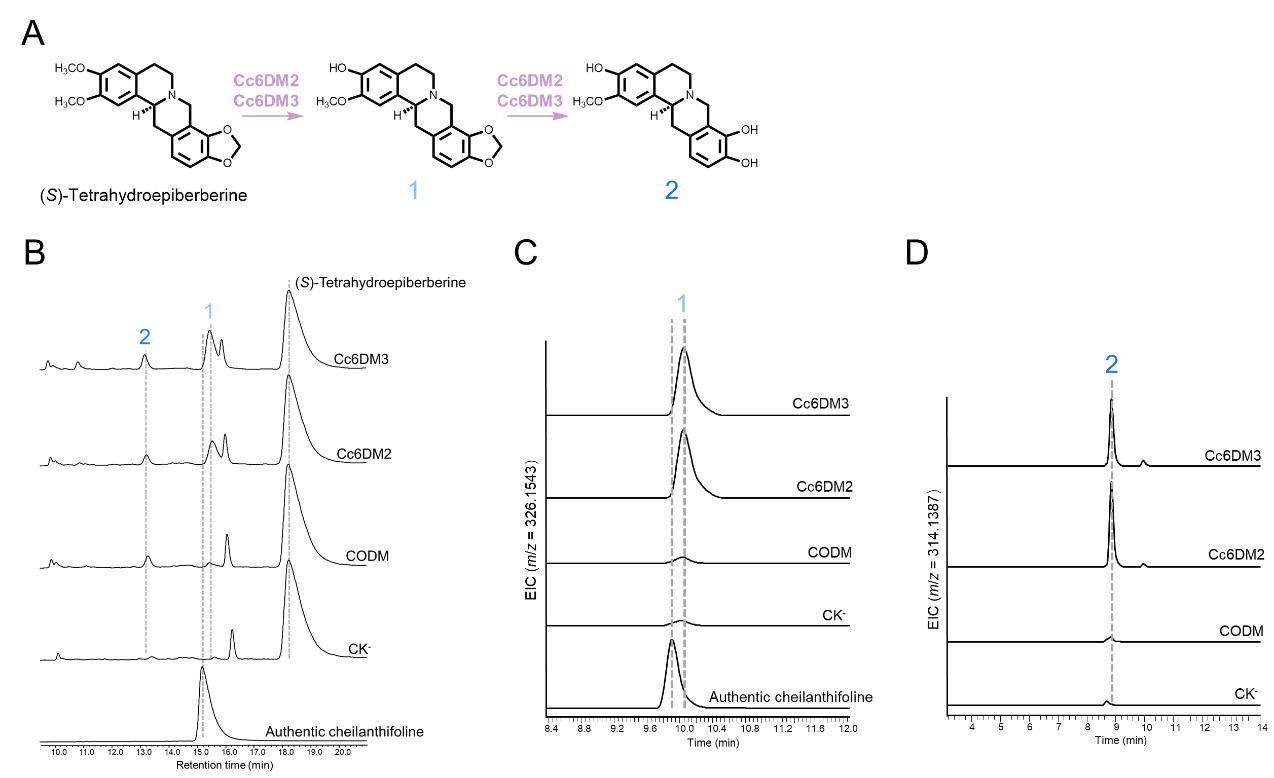


Figure S22. The *O*-demethylation and *O*, *O*-demethylenation of (*S*)-tetrahydroepiberberine.

(A) The proposed demethylation and demethylenation of (*S*)-tetrahydroepiberberine.

(B) HPLC analysis showing the demethylated and demethylenated products of (*S*)-tetrahydroepiberberine catalyzed by CODM, Cc6DM2 and Cc6DM3.

(C) Extracted ion chromatograms according to the *m*/*z* values of demethylated (*S*)-tetrahydroepiberberine, showing the demethylases enzyme assays using (*S*)-tetrahydroepiberberine as substrate. Authentic (*S*)-cheilanthifoline was used for comparison, indicating that the demethylation of (*S*)-tetrahydroepiberberine occurs at C-3.

(D) Extracted ion chromatograms according to the *m*/*z* values of demethylenated (*S*)-tetrahydroepiberberine.


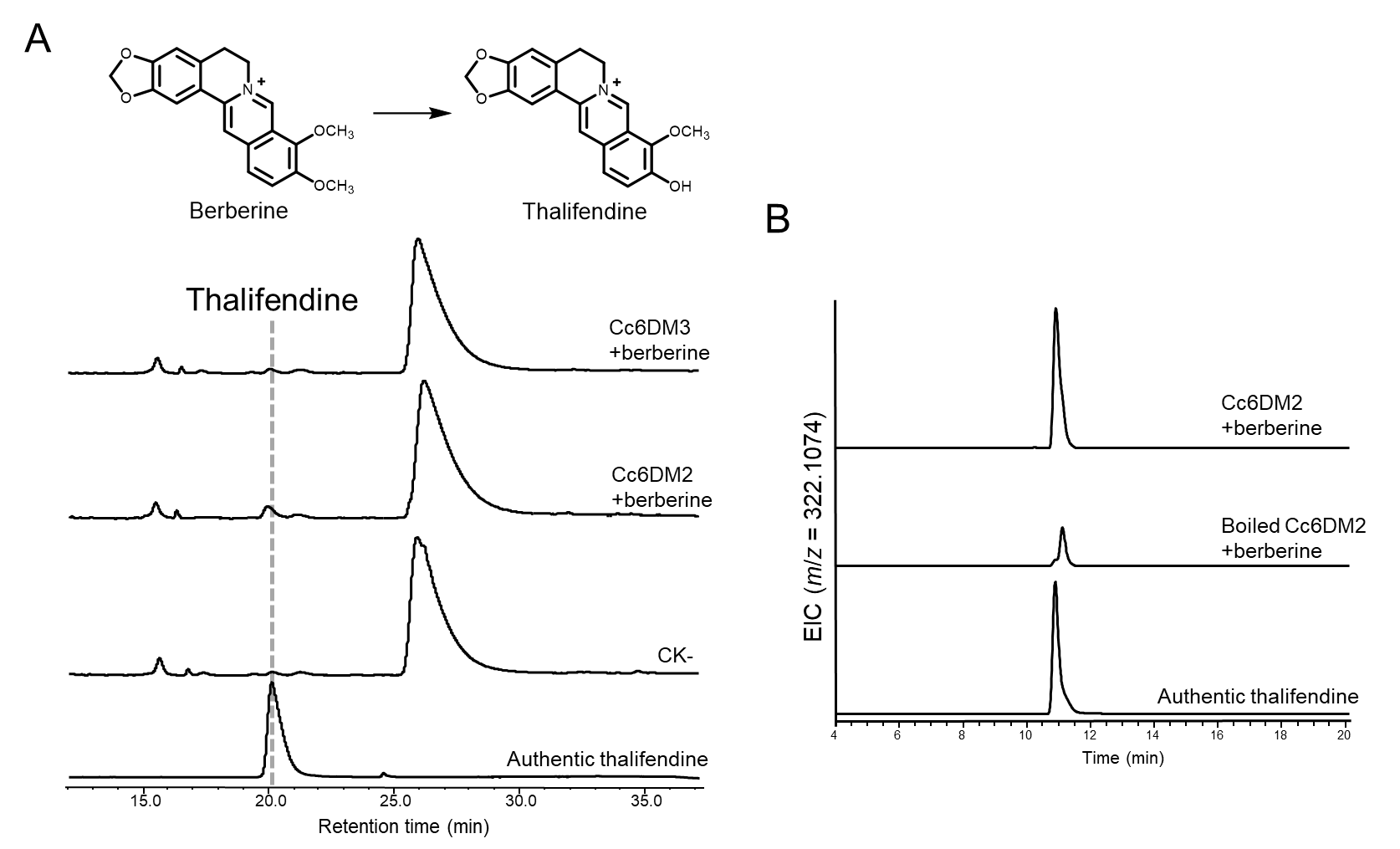


Figure S23. The transformation from berberine to thalifendine under the catalysis of Cc6DM2.

(A) HPLC analysis of the demethylase enzyme assays using berberine as substrate. Trace amount of thalifendine production was observed in the reaction mixture using Cc6DM2.

(B) Extracted ion chromatograms according to the *m*/*z* values of thalifendine, showing the production of thalifendine from berberine catalyzed by Cc6DM2.


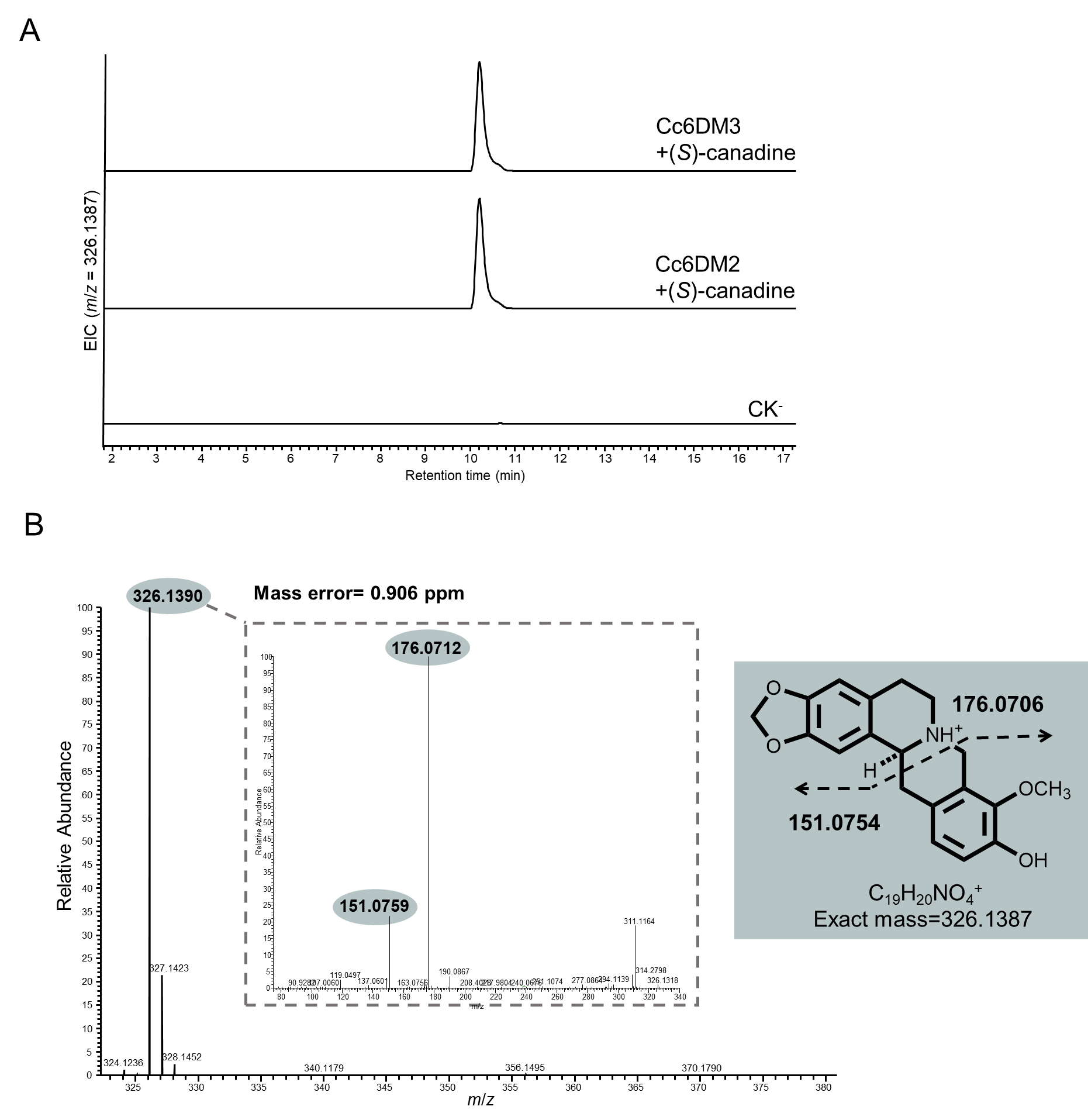


Figure S24. LC-MS analysis of the Cc6DM2 and Cc6DM3 enzyme assays using (*S*)-canadine as substrate.

(A) Extracted ion chromatograms according to the *m*/*z* values of demethylated (*S*)-canadine, showing the enzyme assays of Cc6DM2 and Cc6DM3 using (*S*)-canadine as substrate.

(B) The MS^2^ analysis of the demethylated (*S*)-canadine.

Table S1. The gene ID from reference genome [[3](#_ENREF_3)] and chromosome location of the CYP719 genes characterized in this study.

| Gene name | Gene ID | Chromosome location |
| --- | --- | --- |
| CcCYP719A1 | Cch00017825 | Chromosome 3 |
| CcCYP719A2 | Cch00005300 | Chromosome 4 |
| CcCYP719A3 | Cch00010495 | Chromosome 9 |
| CcCYP719A4 | Cch00017813 | Chromosome 3 |
| CcCYP719A5 | Cch00017821 | Chromosome 3 |
| CcCYP719A6 | Cch00017817 | Chromosome 3 |

Table S2. The estimated ω_0_ and ω_1_ ratios in the branch-site model for the background and foreground lineages respectively in the four site classes. Site class 0 contains sites under purifying selection in both foreground and background lineages, site class 1 contains sites under neutral evolution, site class 2a and 2b contains foregrounds sites under positive selection while background sites under purifying and neutral selection, respectively. ω_1­_>1 indicates that there is significant sign for positive selection in the foreground branch[[4](#_ENREF_4), [5](#_ENREF_5)].

| site class | 0 | 1 | 2a | 2b |
| --- | --- | --- | --- | --- |
| Proportion | 0.64162 | 0.13181 | 0.18795 | 0.03861 |
| background ω­_0_ | 0.12197 | 1.00000 | 0.12197 | 1.00000 |
| foreground ω_1_ | 0.12197 | 1.00000 | 1.97226 | 1.97226 |

**Supplemental references**

1. Morris, J.S. and P.J. Facchini, *Molecular origins of functional diversity in benzylisoquinoline alkaloid methyltransferases.* Frontiers in plant science, 2019. **10**: p. 1058.

2. Li, Y., et al., *Over 100 million years of enzyme evolution underpinning the production of morphine in the Papaveraceae family of flowering plants.* Plant communications, 2020. **1**(2): p. 100029.

3. Liu, Y., et al., *Analysis of the Coptis chinensis genome reveals the diversification of protoberberine-type alkaloids.* Nature communications, 2021. **12**(1): p. 1-13.

4. Yang, Z., W.S. Wong, and R. Nielsen, *Bayes empirical Bayes inference of amino acid sites under positive selection.* Molecular biology and evolution, 2005. **22**(4): p. 1107-1118.

5. Yang, Z., *PAML 4: phylogenetic analysis by maximum likelihood.* Molecular biology and evolution, 2007. **24**(8): p. 1586-1591.
